# Tissue Engineering In Vitro Leaflet- and 3-Dimensional Printing-Based Implant Prototypes for Infant Mitral Valve

**DOI:** 10.34133/bmef.0159

**Published:** 2025-08-07

**Authors:** Martha I. González-Duque, Arielle Breuninger, Frédéric Leis, Julio B. Michaud, Shaginth Sivakumar, Vincent Pautu, Marisa E. Jaconi, Marc Jobin, Adrien Roux

**Affiliations:** ^1^ Tissue Engineering Laboratory, Bioengineering Group, HEPIA HES-SO University of Applied Sciences and Arts Western Switzerland, Geneva, Switzerland.; ^2^ Tissue Engineering Group, Departamento de Farmacia, Facultad de Ciencias, Universidad Nacional de Colombia, Bogotá 111321, D.C., Colombia.; ^3^ Biomedical Sciences Group, Department of Medicine, Universidad Antonio Nariño, Bogotá D.C., Colombia.; ^4^ Department of Basic Neurosciences, Faculty of Medicine, University of Geneva, Geneva, Switzerland.; ^5^ Materials, Optics and Nanotechnology Group, HEPIA HES-SO University of Applied Sciences and Arts Western Switzerland, Geneva, Switzerland.

## Abstract

**Objective:** This study engineers leaflet- and 3-dimensional (3D) printing-based implant prototypes for infant mitral valve repair via in vitro cultured mesoangioblasts isolated from the human fetal aorta (AoMAB). **Impact Statement:** Ultrahigh-molecular-weight polyethylene (UHMWPE) coatings, as well as 3D-printed gelatin methacrylate (GelMA) hydrogels for implants, represent new possibilities for devices used in mitral valve repair. **Introduction:** Mitral valve prolapse (MVP) repair in pediatric patients is challenging due to somatic growth, patient–prosthesis mismatch, reinterventions, infections, and thromboembolism. Tissue-engineered heart valves (TEHVs) offer potential solutions through conventional and 3D printing biofabrication. **Methods:** Four materials are evaluated: UHMWPE, UHMWPE coated with polyvinyl alcohol (PVA), UHMWPE coated with PVA and collagen, and 3D-printed GelMA hydrogels. The prototypes are characterized for micro/nanostructural, physicochemical (degradation, contact angle, Fourier transform infrared spectroscopy), and mechanical properties (simple strength tests, dynamic mechanical analysis) and assessed for cytocompatibility using AoMAB cells. A 3D printing mitral valve prototype is analyzed via immunostaining. **Results:** Results highlight UHMWPE coated with PVA and collagen as the most promising, with degradation (7.30 ± 18.71%), a hydrophilic contact angle (26.13 ± 1.45°), and biocompatibility (177.04 ± 68.92% viability). GelMA prototypes show superior viability (216.77 ± 77.69%) and scalability for 3D printing. **Conclusion:** UHMWPE coated with PVA and collagen and GelMA demonstrate strong potential for TEHVs, with AoMAB cells facilitating 3D culture and future personalized pediatric applications. Further in vitro validation and thrombogenicity assessments are needed.

## Introduction

Mitral valve prolapse (MVP) is a common congenital valve anomaly and the most frequent indication for mitral valve surgery [1]. The gold standard treatment for mitral valve disease is mitral valve replacement with an implant; however, it poses a substantial challenge for pediatric surgical intervention due to the fragile, immature, and small nature of the mitral valve mainly in neonates [2]. Additionally, there are difficulties in obtaining prostheses of appropriate sizes. Pediatric patients must use anticoagulants for extended periods, and their long-term outcomes, as well as the high risks associated with repeated surgical interventions, are less favorable compared to the adult population [1].

Despite continuous improvements of surgical therapeutic approaches with mechanical or bioprosthetic implants, the mortality rate remains high: Approximately 17.9% to 28.6% of patients do not survive 7 years postsurgery [3]. These unsuccessful cases often stem from complications such as thromboembolism, immune rejection, calcification, implant degradation, and infection [4].

Consequently, the main challenges in improved valve implant design for infants are related to maintaining synchronous growth of the implant, size, resistance to infections or thrombosis, as well as durability to avoid surgical reintervention, advancements in materials with antithrombogenic coatings, and the design of small-scale drug delivery systems for transcatheter valves, tailored to patient-specific needs [5]. This therapeutic approach relies on personalized designs that are anatomically adapted to the patient to avoid patient–prosthesis mismatch (PPM) associated with somatic growth. The issue arises from the limited tissue repair-focused therapeutic alternatives, which are specifically designed to address the unique needs of pediatric patients.

Tissue engineered heart valves (TEHVs) seek to mimic native mitral valve tissue and address tissue needs through their 3-dimensional (3D) structure, in which each design parameter corresponds to a specific physiological need. This objective is achieved by selectively incorporating cells, biomaterials, and bioactive factors while considering biomaterial characteristics, valve development techniques, cell origin bioactive factors involved in cell differentiation, and the mechanical properties of the implant due to crosslinking and other processes. Specifically, previous studies have proposed applying valvular interstitial cells (VICs) and valvular endothelial cells (VECs) directly onto a scaffold to promote proliferation and the formation of native tissue ECM. Other studies have utilized induced pluripotent stem cells (iPSCs) and genome editing techniques [6].

Isolating primary human VECs and VICs is challenging, and their proliferative capacity is limited. Therefore, exploring alternative cell types for bioprinting is essential. One such promising candidate for cardiovascular applications and use in tissue-engineered heart valves (TEHVs) is mesoangioblasts isolated from the human fetal aorta (AoMABs) [7]. These cells are characterized as a subpopulation of pericytes or vessel-associated stem/progenitor cells capable of self-renewal and differentiation into various mesoderm cell types, including skeletal and cardiac muscle. The high proliferation rate and differentiation capabilities of AoMAB cells, along with their expression of common mesenchymal phenotypic markers shared with VICs, as well as their ability to differentiate into osteogenic, myofibroblastic, and chondrogenic lineages, make them well suited for in vitro studies of cellularized heart valve implants [7].

The scaffold manufacturing process is also a key component of valve implant design. Tissue engineering via 3D bioprinting allows cellular hydrogel constructs to be made with patient-specific anatomical geometry and mechanical properties with customized printability, shape fidelity, and bioactivity [8]. In the pediatric field, advancements in implant size, the use of autologous or allogenic cells, and the implementation of personalized designs enhance biocompatibility, reduce mismatches, and incorporate high-quality control measures to facilitate scalability with limited environmental impact [9]. However, the disadvantages are primarily associated with the high costs of bioprinting processes today, as well as the maneuverability of the delivery systems.

The aim of this study was to engineer and characterize leaflet- and 3D printing-based implant prototypes for infant mitral valve repair using in vitro cultured AoMAB cells.

Consequently, the selection of materials for the leaflet and implant prototype is of paramount importance in both the bioprinting process and conventional manufacturing. Some of the most prominent materials in use for constructing TEHV scaffolds are synthetic biopolymers and naturally occurring materials. In the case of the former, popular polymers include polyglycolic acid, poly(lactic-co-glycol) (PLGA), and polyhydroxyalkanoates, whereas alternatives such as collagen, elastin, alginate, cellulose, gelatin, hyaluronic acid, chitosan, and keratin are widely used materials for the latter [10].

For instance, previous studies have investigated the volumetric bioprinting properties of gelatin methacrylate (GelMA), a biocompatible and osmotic material derived from porcine gelatin, which exhibits optimal degradation time and cytocompatibility and supports cell migration [11]. Methacrylate groups incorporated into its structure enable crosslinking with a photoinitiator under ultraviolet light exposure, thereby enhancing its structural stability while preserving a consistent refractive index during polymerization [12]. Similarly, polyvinyl alcohol (PVA) is a synthetic polymer with high clinical promise in TEHV applications because it is highly compatible with both a hydrogel and a freeze-dried material, is characterized by physical cross-linking, and is hydrophilic. Given that hydrophilicity has been linked to biocompatibility, blending PVA with other molecules has been shown to improve cell adhesion and growth in vitro [13]. Collagen is also an encouraging material due to its biocompatibility, low immunogenicity, and potential for controlled and customizable biodegradation [14].

Nonetheless, one of the most compelling materials currently being explored for TEHVs is ultrahigh-molecular-weight polyethylene (UHMWPE). A synthetic polymer, UHMWPE, possesses a uniquely favorable balance between a low coefficient of friction and high wear and fatigue resistance [15]. Furthermore, the material is flexible, antithrombogenic, antibacterial, and biocompatible in cardiovascular and orthopedic applications [16]. Several limitations of UHMWPE particles exist—including relatively low thermal stability and low load-bearing capacity—although incorporating UHMWPE-based micro- and nanocomposites could be an effective solution for addressing these mechanical shortcomings [15].

These implant base materials can be implemented in the final design through a variety of methods, including laser cutting of desired shapes or through 3D printing techniques. Specifically, recent studies have described the successful use of 3D bioprinting via extrusion, laser, robotic arm, and acoustic techniques or through volumetric additive manufacturing with photosensitive resin [17].

The design possibilities in bioprinting vary greatly from standard mass-produced prototypes to patient-specific implants. However, standard bioprinting procedures could be effectively applied in cases such as printing of Ozaki templates for aortic valve reconstruction, where autologous pericardium obtained surgically must have defined, measured values cut for implantation [18].

This study explores leaflet- and 3D printing-based prototypes using UHMWPE, UHMWPE-PVA, UHMWPE-PVA + collagen, and GelMA hydrogels. These materials were evaluated for their structural, physicochemical, mechanical, and cytocompatibility properties with AoMAB cells. A 3D-printed valve prototype was also analyzed by immunostaining to assess biological integration.

## Results

### Prototypes and validation

**Fig. 1.**
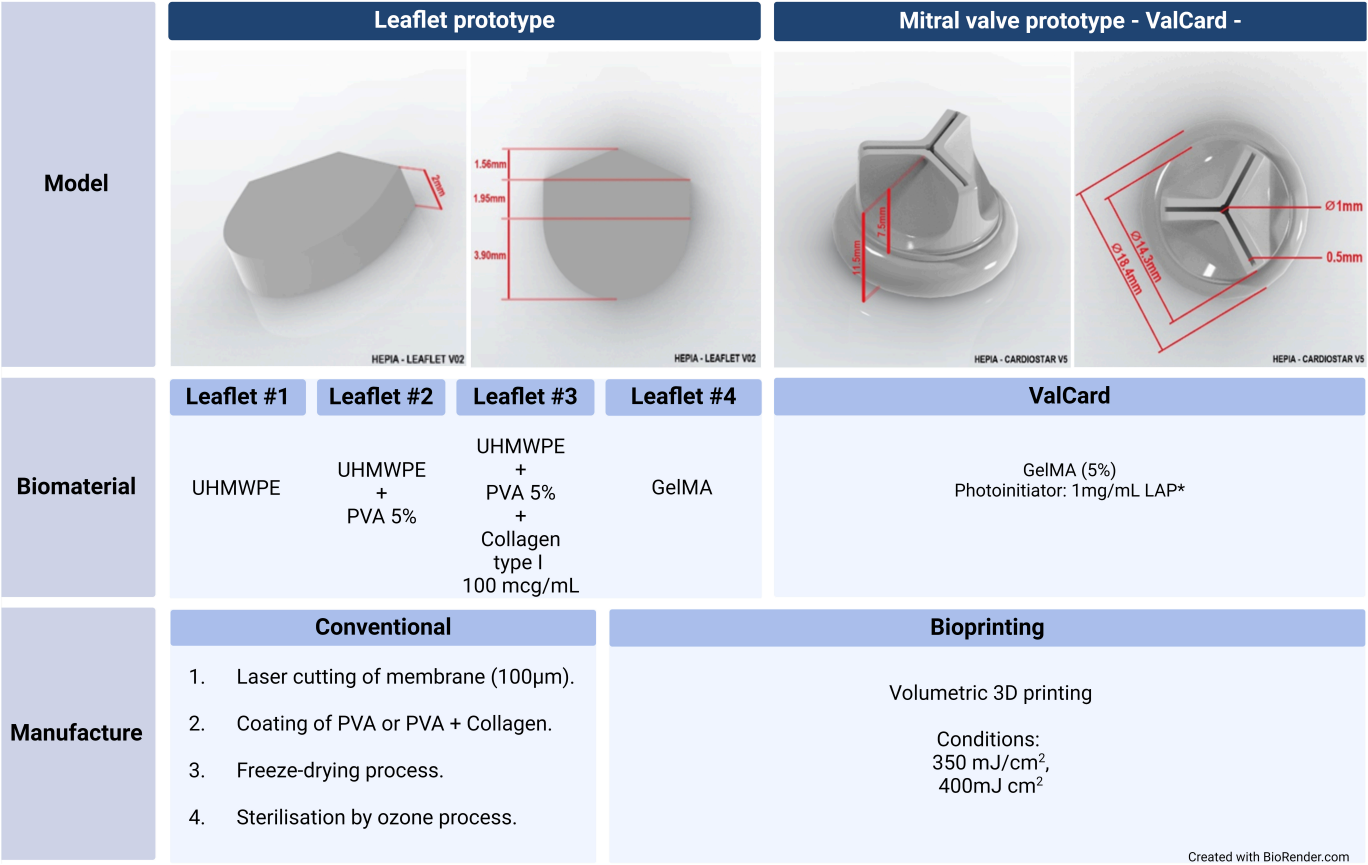
Design and biomaterials for leaflets and mitral valve prototypes.

We generated 4 leaflet prototypes with the same dimensions (Fig. 1). Leaflet #1 (control) was made of 100-μm UHMWPE; leaflets #2 and #3 were developed via conventional coating and freeze-drying techniques using UHMWPE coated with PVA and PVA + collagen, respectively. Leaflet #4 was constructed from 3D-printed GelMA. For all the scaffolds, the leaflets were 10 mm in diameter and adhered to the Ozaki model for leaflet-based implant design [19,20], with modifications for in vitro culture in 24- and 48-well plates. The mitral valve prototype model—ValCard—was 3D printed for postprinting culture on 12-well plates. This 3D culture design was validated through established design parameters based on material biocompatibility and mechanical properties (Table S1).

### Characterization of the leaflet prototypes

We characterized the 4 leaflet prototypes according to the following properties: microstructure, physicochemistry, mechanics, nanostructure, and cytocompatibility. Valcard, the newly engineered mitral valve prototype proposed in this study for implant application, was evaluated with AoMAB cells in culture.

#### Microstructural study

**Fig. 2.**
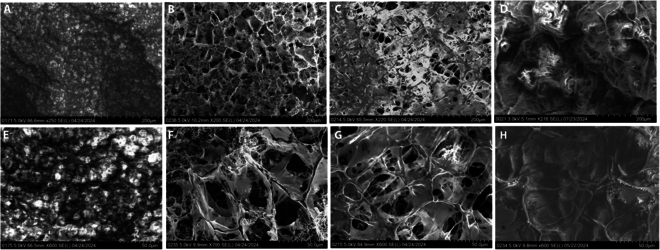
ESEM images of the surface of the leaflet prototypes. (A and E) Leaflet #1 UHMWPE. (B and F) Leaflet #2 UHMWPE + PVA 5%. (C and G) Leaflet #3 UHMWPE + PVA 5% + 100 μg/ml collagen. (D and H) Leaflet #4 GelMA 7.5% w/v. Scale bars, 200 μm (A to D) and 50 μm (E to H).

Microstructural characterization results obtained from surface of leaflets #1 to #4 via environmental scanning electron microscopy (ESEM) are presented in the microstructural characterization in Fig. 2. Leaflet #1 exhibited ill-defined surface pores and UHMWPE fibers; in leaflets #2 and #3, well-defined pores with structured multiaxial fibers were observed. Given the hydrogel structure of GelMA for leaflet #4, these samples were subjected to a freeze-drying process for observation via ESEM, and analysis demonstrated evidence of well-defined pores as well as laminar edges without interconnection on the surface.

Table 1 reports that the pore size was determined via 2 different techniques with ImageJ software. The pore area results calculated via technique 1, which relies on a manual selection process, closely align with the trends demonstrated by the pore diameter results. Furthermore, there was statistically significant difference (*P* ≤ 0.05) in pore diameter that was detected between leaflet #1 (11.26 ± 2.60 μm) and leaflets #2 (50.37 ± 18.96 μm) and #3 (44.92 ± 17.18 μm). Similarly, leaflet #4 (17.82 ± 5.84 μm) was significantly different from leaflets #2 and #3.

**Table 1.** Pore size of the leaflet prototypes. The values are presented as the means ± SDs: *P* ≤ 0.05: pore size (area). Technique 1: (#1 to #2; #1 to #3; #2 to #4; #3 to #4), *n* = 50 pores. *P* ≤ 0.05: pore size (area). Technique 2: (#1 to #2; #2 to #3), *n* = (#1 = 575 pores; #2 = 2,554 pores; #3 = 1,206 pores; #4 = 12 pores). *P* ≤ 0.05: pore size (diameter). Technique 1: (#1 to #2; #1 to #3; #2 to #4; #3 to #4), *n* = 50 pores.

Characteristic of leaflet	#1 UHMWPE	#2 UHMWPE + PVA	#3 UHMWPE + PVA + collagen type I	#4 GelMA
Pore size (area)	10.30 ± 4.82	228.32 ± 182.39	185.91 ± 148.38	29.55 ± 22.94
Pore size (area) Technique 2 (μm^2^)	16.44 ± 27.15	28.64 ± 52.21	22.34 ± 43.07	24.71 ± 23.78
Pore size (diameter) Technique 1 (μm)	11.26 ± 2.60	50.37 ± 18.96	44.92 ± 17.18	17.82 ± 5.84

The pore sizes of leaflets #2 and #3 were indicative of macropores; however, while the effective pore size obtained in these prototypes was found to be less than 100 μm, this value was much greater than the average pore size obtained from the uncoated UHMWPE particles (leaflet #1). Technique 2 calculates the pore size (area only) via a threshold-based automatic detection program capable of detecting pores of a far greater size range, specifically very small pores present in the material. Consequently, these results revealed high standard deviations in pore size for each leaflet, with statistical significance (*P* ≤ 0.05) observed only between leaflets #1 (16.44 ± 27.15) and #2 (28.64 ± 52.21), as well as between leaflets #2 (28.64 ± 52.21) and #3 (22.34 ± 43.07). Further details on the results and pore classification are reported in Table S2.

#### In vitro biodegradation

**Fig. 3.**
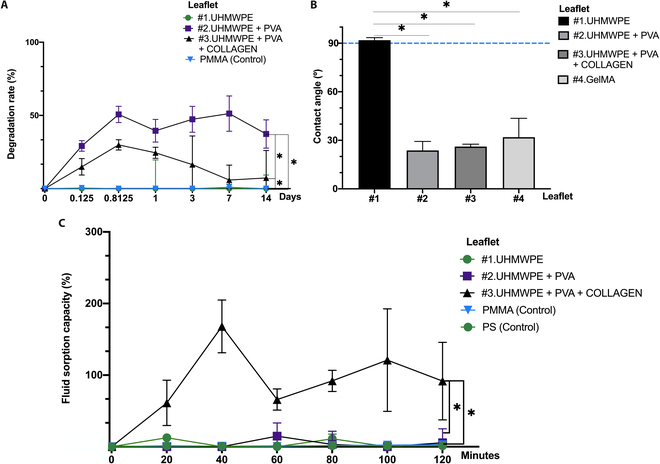
Assessment of degradation rate, contact angle, and fluid sorption capacities of leaflet #1 UHMWPE, leaflet #2 UHMWPE + PVA 5%, leaflet #3 UHMWPE + PVA 5% + 100 μg/ml collagen type I, and leaflet #4 GelMA 7.5% w/v. (A) Degradation rate for each leaflet prototype (%). Control: PMMA. *n* = 3; mean ± SD. Significant differences: *P ≤ 0.05. (B) Contact angle (°), *n* = 4; mean ± SD. Significant differences: *P ≤ 0.05. (C) Fluid sorption capacity (%) percentage. Control: PMMA and PS. *n* = 3; mean ± SD. Significant differences: *P ≤ 0.05.

The percentage of degradation by collagenase IV was determined at 6 different time points over the course of 14 d. Overall degradation by day 14 was measured for leaflets #1 (7.87 ± 17.14%), #2 (37.31 ± 9.66%), and #3 (7.30 ± 18.71%). Leaflet #4 was not evaluated due to structural limitations in conducting the assay on a hydrogel. Polymethylmethacrylate (PMMA) was utilized as a negative control sample (−0.02 ± 1.19% at 14 d). As outlined in Fig. 3A, significant differences (*P* ≤ 0.05) were observed on day 14 between the following prototypes: leaflets #2 and PMMA, leaflets #3 and PMMA, leaflets #1 and #3, and leaflets #1 and #2. Furthermore, the addition of collagen to a PVA-only leaflet coating resulted in an overall lower level of leaflet degradation. Values less than 0 were reported as equal to 0; specific test data are recorded in Figs. S2 and S3.

#### Contact angle

Leaflet hydrophilicity was quantified via contact angle measurements with deionized water, as shown in Fig. 3B. Leaflet #1 presented a far greater contact angle than the other leaflet samples did, with a value around the hydrophilicity limit (91.90 ± 1.57°). The UHMWPE-based leaflets with coatings exhibited significantly smaller contact angles, at 23.74 ± 5.57° for leaflet #2 and 26.13 ± 1.45° for leaflet #3. Leaflet #4 also had a relatively low contact angle (31.90 ± 11.70°), and these latter 3 materials were all found to be hydrophilic. Significant differences (*P* ≤ 0.05) between leaflet #1 and leaflets #2, #3, and #4 were detected.

#### Fluid sorption capacity

The fluid sorption capacity of each leaflet was evaluated to simulate implant interactions with native tissue in aqueous medium (Fig. 3C). The samples were analyzed over the course of 2 h (with measurements every 20 min). Values less than 0 were approximated to 0 on the graph (Fig. S4). At 120 min, leaflet #3 had the greatest percentage change in weight due to sorption (91 ± 93.81%) and was significantly different (*P* ≤ 0.05) from all the other leaflets and the controls. The average fluid sorption capacity for leaflet #1 (3.70 ± 6.41%), leaflet #2 (5.55 ± 33.67%), and the negative controls polymethyl methacrylate (PMMA) (2.70 ± 0.48%) and polystyrene (PS) (1.19 ± 0.59%) generally displayed negligible sorption values (see Figs. S4 and S5).

#### Fourier transform infrared spectroscopy

**Fig. 4.**
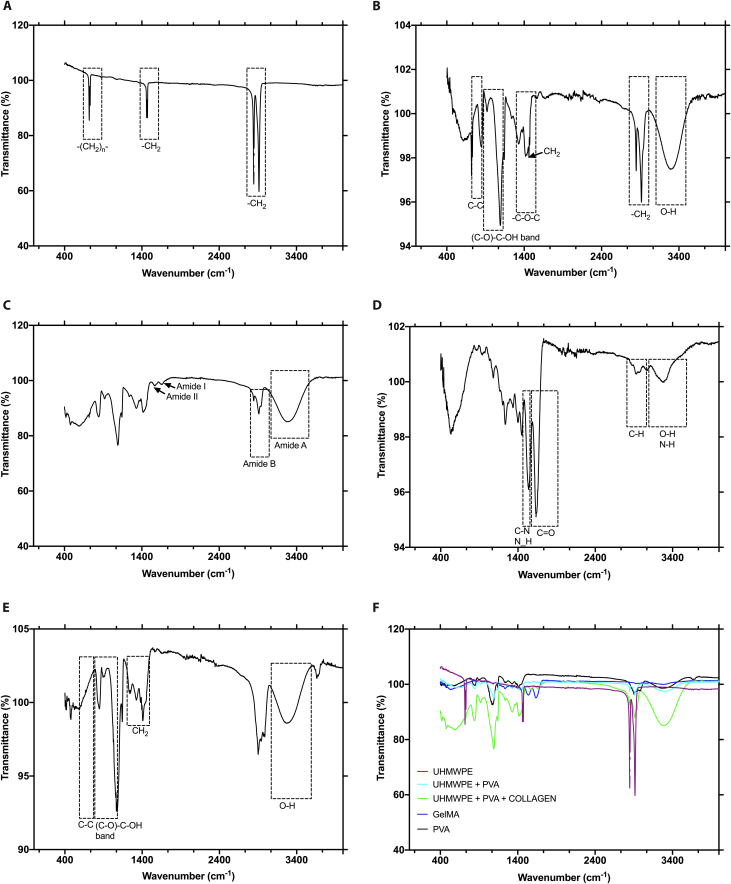
FTIR analysis of the leaflet prototypes. *n* = 4. (A) Leaflet #1 UHMWPE. (B) Leaflet #2 UHMWPE + PVA 5%. (C) Leaflet #3 UHMWPE + PVA 5% + 100 μg/ml collagen type I. (D) GelMA 7.5% w/v. (E) Control (PVA). (F) Spectra of leaflets #1 to #4 and the control.

We determined the chemical composition of each leaflet prototype using Fourier transform infrared (FTIR) analysis as depicted in Fig. 4. The leaf #1 spectra presented characteristic peaks at 2,914 cm^−1^ and 2,847 cm^−1^ (products of polyethylene oxidation as well as hydrogen bonded and nonbonded hydroperoxide), 1,462 cm^−1^ (vibrations for ethers and other groups), and 719 cm^−1^ (absorption for transethylene groups) (see Fig. 4A). Leaflet #2 exhibited spectra consistent with those of UHMWPE and PVA, with characteristic peaks at 834 cm^−1^ (C–C), 1,087 cm^−1^ [(C–O)–C–OH], 1,318 cm^−1^ (-C–O–C), and 3,260 cm^−1^ (OH stretching) (see Fig. 4B). Leaflet #3 spectra presented peaks characteristic of UHMWPE, PVA, and collagen type I at 3,247 cm^−1^ (amide A), 2,905 cm^−1^ (amide B), 1,628 cm^−1^ (amide I), and 1,531 cm^−1^ (amide II) (see Fig. 4C). Leaflet #4 displayed absorption spectra expected for GelMA, with peaks at approximately 3,346 cm^−1^ (O–H and N–H stretching) between 2,800 cm^−1^ and 3,100 cm^−1^ (C–H groups), 1,631 cm^−1^ (amide I), and 1,531 cm^−1^ (amide II) (see Fig. 4D). PVA was used as a positive control (see Fig. 4E) and exhibited typical absorption peaks at approximately 834 cm^−1^ (C–C), 1,077 cm^−1^ [(C–O)–C–OH], 1,318 cm^−1^ (-C–O–C), and 3,260 cm^−1^ (OH group stretching). Figure 4F shows all the leaflet prototypes (#1 to #4), as well as the PVA, which presented typical absorption peaks according to their respective chemical compositions.

#### Simple mechanical strength test

We performed tensile strength mechanical testing for each leaflet to assess prototype strength and elasticity. We found that neither the PVA nor the PVA + collagen type I coatings provide a statistically significant (*P* ≥ 0.05) effect on the maximum force, tensile strain, elastic modulus, force break, or force at 0.2% plastic strain for the UHMWPE-based leaflets (see Table 2). The elastic modulus decreased slightly for leaflet #2, which was likely attributed to the PVA coating.

**Table 2.** Simple mechanical strength test. The values are presented as the means ± SDs: *P* ≥ 0.05 (ns) indicates no significant differences in mechanical properties between leaflets #1, #2, and #3.

Mechanical properties	#1 UHMWPE	#2 UHMWPE + PVA	#3 UHMWPE + PVA + collagen type I
Maximum force (*N*)	3.622 ± 0.105	3.612 ± 0.124	3.582 ± 0.268
Tensile strain (displacement) at break (standard) (%)	9.175 ± 0.884	9.45 ± 1.623	9.041 ± 1.850
Elastic modulus (GPa)	0.190 ± 0.021	0.177 ± 0.025	0.179 ± 0.022
Force break (*N*)	3.601 ± 0.111	3.59 ± 0.121	3.557 ± 0.271
Force at 0.2% plastic strain (*N*)	0.907 ± 0.154	0.850 ± 0.166	0.842 ± 0.139

#### Dynamic mechanical analysis

We performed dynamic mechanical analysis (DMA) on leaflets #1 to #4 (Fig. S6) in the temperature range of 37.5 to 39.5 °C and assessed the storage modulus (*E*′), loss modulus (*E*″), and loss factor (tan δ). For each leaflet, the initial *E*′ was found to be approximately 2 to 35 MPa, whereas the initial *E*″ was approximately 2 to 6 MPa. Furthermore, the *E*′ curves for leaflets #2 and #3 exhibited a stability in this temperature range. The curve of tan δ was indicative of a specific shape that corresponded to the energy transitions among the layers of these composite materials for leaflets #2 and #3. Leaflet #4, however, exhibited an upward *E*′ curve with a peak at 35 MPa and generally displayed mechanical behavior distinct from those of the other 3 thermoplastic leaflets. As UHMWPE-based leaflets, their respective values for *E*′ were instead relatively low and stable.

#### Nanostructural characterization

Additionally, we evaluated the nanostructural properties of the leaflets (Fig. S7A to D). The force maps revealed differences among the 4 different leaflets, with leaflet #1 being more homogeneous than leaflets #3 and #4. Leaflet #1 demonstrated a topography typical of UHMWPE—given the presence of elevations on its surface in a structured manner—while elevations in the topography of leaflet #2 were largely irregular. The surface of leaflet #3 was regularly patterned, although with a defined rough appearance. Finally, leaflet #4 exhibited a smooth nanoscale topography with a concave curvature that was generally void of major elevations (Fig. S7E to H). The nanoscale mechanical properties varied significantly among the prototypes, with leaflet #4 possessing the lowest elastic modulus, and the biomaterial coatings greatly reduced the elastic modulus of the UHMWPE-based leaflets. Variations in adhesion and stiffness were also observed in this characterization, with values once again resting notably lower for leaflet #4.

#### Cytocompatibility of the leaflet prototype

##### Viability test

**Fig. 5.**
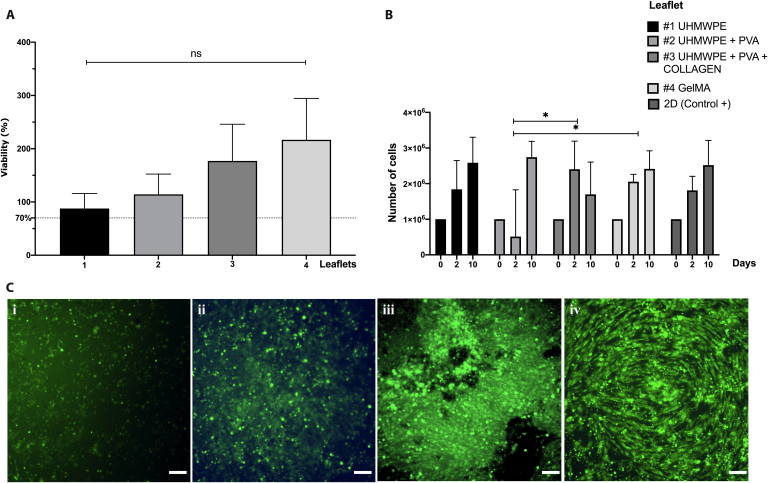
Cytocompatibility of AoMAB cells within leaflets. (A) Cell viability percentage (%), *n* = 3, mean ± SEM: *P* ≥ 0.05 (ns) shows no significant difference. Leaflet #1 UHMWPE; leaflet #2 UHMWPE + PVA; leaflet #3 UHMWPE + PVA + collagen type I; leaflet #4 GelMA. (B) AoMAB proliferation on leaflets. *n* = 3, mean ± SD: *P* ≥ 0.05 (ns) indicates no significant differences. Leaflet #1 UHMWPE; leaflet #2 UHMWPE + PVA; leaflet #3 UHMWPE + PVA + collagen type I; leaflet #4 GelMA; 2D AoMAB cell culture was used as a positive control (+). Time (0, 2, and 10 d). (C) Cell adhesion over leaflets (5 d). (i) Leaflet #1 UHMWPE. (ii) Leaflet #2 UHMWPE + PVA. (iii) Leaflet #3 UHMWPE + PVA + collagen type I. (iv) Leaflet #4 GelMA.

We evaluated the viability of AoMAB cells for each leaflet to be above 70% among all prototypes (see Fig. 5A), a parameter that highlights their respective compatibility with these cells. The highest viability was obtained by leaflet #4 (GelMA), at 216.77 ± 77.69%, followed by leaflet #3, at 177.04 ± 68.92%. Leaflets #2 and #1 presented viabilities of 114.33 ± 38.05% and 87.79 ± 28.11%, respectively. No significant differences were found among the prototypes.

##### Cell proliferation

We assessed the cell proliferation chart for each leaflet (Fig. 5B), with AoMAB cells in conventional 2D culture as a control. The samples were evaluated at 3 different time points (0, 2, and 10 d). Maximum proliferation was generally obtained at 10 d postseeding. Leaflet #2 presented the greatest variability in average cell count as a function of time, whereas the most stable prototype over time was leaflet #3.

##### Cell adhesion

Adhesion testing was used to quantify the rate of cell adhesion to the leaflet scaffolds 5 d after seeding. Figure 5C displays the different cell adhesion patterns associated with each type of leaflet. While leaflets #1 and #2 exhibited a superficial pattern of adhesion, leaflet #3 demonstrated greater adhesion to the surface of the material, and leaflet #4 generated a concentric pattern. These patterns can be attributed to cell proliferation trends induced by a specific leaflet biomaterial. Fluorescence intensity quantification for each leaflet is presented in Fig. S8.

#### Cytocompatibility of the Valcard prototype of mitral valve implant

**Fig. 6.**
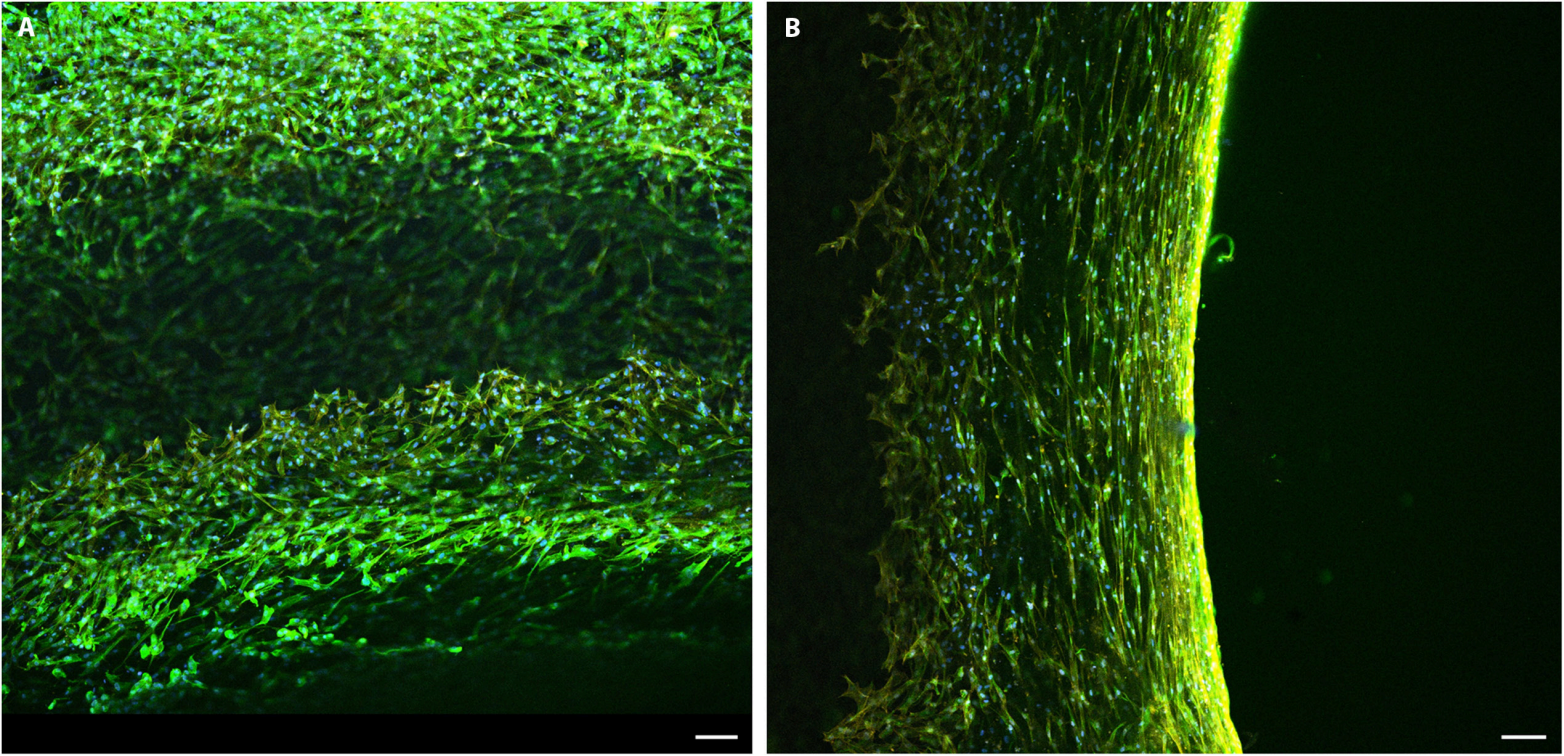
Immunostaining analysis of AoMAB cells on the mitral valve implant prototype Valcard. In vitro culture of the 3D structure implant prototype with GelMA was performed for 14 d. Green, vimentin; blue, nucleus. Scale bar, 100 μm. (A) Superficial view of the medial surface of the prototype. (B) Superficial view of the lateral surface of the prototype.

Following the evaluation of the leaflets, we adjusted the design parameters of the Valcard implant to account for pediatric growth, valve implant safety, and biocompatibility. These adjustments were aimed at optimizing cell viability, proliferation, and adhesion, as well as enhancing the biomaterial’s versatility for 3D design. GelMA was chosen as the biomaterial for the prototype due to its potential to enable scalability toward the development of a complete valvular model through volumetric bioprinting, followed by seeding with AoMAB cells. Cell viability was evaluated in transparent 18.4-mm-diameter Valcard implants at 14-d of culture in 12- and 24-well plates (Fig. 6). Confocal, MesoSPIM, and light sheet microscopy confirmed cell proliferation around the entire implant surface (Movies S1 to S3).

## Discussion

This study evaluated a range of materials as leaflet implant candidates for infant mitral valve repair. A special consideration was thus taken for designing a structure that could synchronously grow and adapt to a growing child and demonstrate durability and strong mechanical properties [21,22]. We present here 4 leaflet designs for partial mitral valve repair and a mitral implant for complete valve replacement, using 2 different techniques. (a) The conventional freeze-drying process consists on freezing followed by solvent sublimation of solvent to obtaining a porous structure, but these have limitations for custom personalized 3D designs and mechanically resistant, such as for the full implant. Nevertheless, this method is easily manageable for the development of leaflets of different sizes for in vitro culture. (b) The 3D printing technique in volumetric bioprinters has the advantages of precision in size and complex and customized designs, as well as optical material transparency facilitating the evaluation of cytocompatibility by different advanced microscopy techniques [17].

A design for in vitro culture and growth of a leaflet or a complete valve implant would represent an advance for translational medicine in the pediatric field [23]. Current leaflet designs aim at autologous pericardial implants [18], yet the use of high-performance biomaterials such as UHMWPE [24] could be an alternative provided an appropriate coating that improves their profile [15], such as the leaflets obtained here that were characterized on the basis of their microstructural, nanostructural, physicochemical, and mechanical properties as well as cytocompatibility.

As the tested leaflet materials can be classified into 2 distinct categories—UHMWPE-based polymers and GelMA hydrogels—their material characteristics, the respective strengths, and even the characterization tests had distinct and variable feasibilities.

Multiple assays require GelMA to be lyophilized (used for dried bulk hydrogel) prior to experimentation (SEM, porosity), which may have affected the surface appearance of the material and pore sizing, porosity, and interconnectivity. Visually, the pore size appears to form a nonporous structure [25], with pores potentially smaller than 10 μm, thereby limiting cell infiltration. Additionally, GelMA (leaflet #4) precluded from the characterization tests of fluid sorption, degradation, and traction due to the mechanical strength limitations [26] of the 2-mm-thick construct. However, this restriction is overcome by designing greater thickness and sizes.

Leaflets coated by PVA and PVA + collagen type I improved the pore sizes, thereby acquiring microstructural properties that are more culture friendly. Indeed, the coating can modify the smooth surface and nonpolar functional groups so that UHMWPE regularly has poor adhesion and low interfacial properties [27]; PVA and collagen type I then modify the surface, as visualized by SEM, and introduce polar functional chemical groups and roughness to the surface, as subsequently evidenced by FTIR and nanotopography. This explains the reduction in the contact angle of leaflets #2 and #3 and their sorption fluid capacity [27].

Both techniques of pore analysis—a traditional manual approach and a novel automated method—yield unique strengths and weaknesses. The automatic pore detection technique designed pores as nonelliptical shapes, aiming to approximate pore detection as close to the true shape as possible. This method facilitated a more precise delineation of the pore structures, and the pore size (which is typically determined through standard major and minor axis measurements) could not be calculated [28]. Nonetheless, one of the main advantages of this technique is its significantly faster analysis time. Particularly in samples with a high quantity of small pores, automatic detection is optimal because of both in terms of efficiency and ability to effectively identify small pores scattered throughout a sample. As the range of detectable pore sizes was greater with the automatic detection method, the pore area data results achieved in this manner are more accurate. This conclusion generally falls in line with the measured results, as leaflets #1 and #4—with smaller pore sizes—have areas fairly consistent across both techniques. Measurements for leaflets #2 and #3, meanwhile, are far larger in technique 1, where a high level of small pores is likely not detected manually.

Some limitations remain, for which traditional ellipse methods are preferable. Threshold-based detection is not effective for images with uneven lighting, where shadows may be difficult to distinguish computationally from pores. Therefore, only images of surfaces with generally smooth textures are recommended for this approach based on deep learning. In an image with irregular pores and a count greater than 50, the automated technique 2 could be implemented.

The pore size of scaffold is crucial for promoting migration, cellular infiltration, vascularization, and the effective diffusion of nutrients, oxygen, and waste products [29]. The pore size of the PVA scaffolds or hydrogels has a wide range (50 to 650 μm) according to the results for leaflet #2, with a minimum reduction in leaflet #3 (with collagen type I) but a sufficient size for mesenchymal cells (17.9 to 30.4 μm) [30]. References for GelMA have an average pore size for hydrogels between 5 and 150 μm, according to the results obtained on leaflet #4 [31].

The percentage of degradation obtained at 14 d for UHMWPE is consistent with the resistance of this material to in vivo degradation. Owing to its chemical composition, the degradation rate of leaflet #2, which contains type I collagen, is greater than that of leaflet #3, which is related to the ability of collagenase to hydrolyze peptide bonds [32]. The presence of polar groups, such as hydroxyl groups (-OH) in PVA, particularly in leaflets #2 and #3, indirectly promotes the interaction with collagenase. This effect is attributed to the increased hydrophilicity of the polymer matrix, which facilitates water uptake and swelling and potentially enhances enzymatic interactions with the material surface, although PVA itself is not a natural substrate for collagenase.

The contact angles obtained in the UHMWPE are those expected for this material, which has more hydrophobic characteristics; however, the coating of leaflets #2 and #3 can modify this parameter and improve hydrophilicity by decreasing the contact angle, given the greater presence of polar groups in PVA [27]. This property is also evident in the fluid sorption capacity, which is much greater for the collagen-containing coating of leaflet #3, improving the interaction of the biomaterial with the aqueous medium [33].

FTIR analysis confirmed the chemical composition of each leaflet prototype, which presented typical absorption peaks according to their respective chemical compositions. The mechanical performance of the leaflet prototypes largely corresponded with findings from previous literature [34] and was critical in evaluating the potential of this study’s implant designs for an effective implementation for mitral valve repair. Macroscale mechanics of UHMWPE are complex to express generally, as a specific formulation technique plays a large role in determining these properties, as does the fact that UHMWPE is often utilized as the base of a composite with others. These results are promising and favor the use of implant coatings engineered for biocompatibility while not compromising mechanical properties in the case of PVA + collagen type I. The results for DMA are correlated with leaflets #1 to #3 as thermoplastic materials.

Young’s modulus is markedly different between mechanical stress tests and AFM, as these 2 methods operate on different scales. This is because the nanoindentation technique is not influenced by all components of the sample, allowing for the identification of the modulus of individual components [35]. However, it offers nanostructurally a key piece of data to understand the possible interaction of cells with the surface of each leaflet. Also, given the hydrogel nature of GelMA, adhesion and stiffness data differ significantly from the other prototypes [36]. The differences between leaflets #2 and #3 are striking despite that the PVA coating amount of type I collagen is 100 μg/ml, which is sufficient to change the nanocharacteristics of the leaflet surface and is shown by microstructural results.

AoMAB cells have good performance in viability test in all leaflets evaluated [7]. The fast degradation rate of leaflet #2, with a PVA coating and its hydrophilicity, could cause cells to detach from the surface and affect the cell viability count. We tend to exclude possible contamination issues as not apparent, The different adhesion pattern observed between leaflets #3 and #4 may be associated with variations in pore size. In leaflet #4 (GelMA), cells might encounter initial migration challenges after printing. Despite this, the overall process remains viable, as evidenced in Fig. 5. For this reason, they are preserved as concentric patrons, and the mechanical force inherent for the leaflet may affect the orientation of different molecular and cellular processes at the cellular adhesion interface at the micro- and nanoscale [37].

Finally, leaflet #3 has good microstructural and nanostructural characteristics (pore size), as well as contact angle, fluid sorption capacity, simple mechanical strength test, nanostructural characterization, viability test, and proliferation and adhesion efficiency, and other tests are foreseen in the future such as permeability, thrombogenicity, and in vivo testing in animal models. Similarly, mitral valve implant prototype Valcard needs specific characterization according to the ISO 5840-1:2021 standards [38], given its excellent in vitro performances in this study.

The durability of UHMWPE-based leaflet polymers supports their adaptability for pediatric applications, although this depends on further preclinical studies. However, based on the results presented, it can be hypothesized that these materials may offer potentially reparative performance, with growth dependent on the 3D cell-based construct. This could improve adaptability, although the disadvantage of requiring surgical reinterventions due to somatic growth would remain. In contrast, GelMA-based leaflets may offer a more regenerative approach, as their greater cellular compatibility could promote the generation of consistent valvular tissue that grows with the pediatric patient. Nevertheless, these constructs still present limitations in terms of maneuverability. Therefore, further studies are required.

## Conclusion

This study engineered and characterized leaflets for in vitro culture, with potential applications in pediatric valve repair, utilizing AoMAB cells. Leaflets based on UHMWPE (leaflets #2 and #3) with coatings showed improvements in pore size, hydrophilicity, and cytocompatibility while maintaining their mechanical properties and exhibiting low degradation rates. Among these, leaflet #3 (UHMWPE coated with PVA and collagen type I) demonstrated the best performance, with in vitro degradation at 14 d (7.30 ± 18.71%), a hydrophilic contact angle (26.13 ± 1.45°), and adequate cell viability at 10 d (177.04 ± 68.92%). Leaflet #3 holds potential as a long-term and effective treatment option, inspired by the Ozaki model for valvular repair, with applications in the pediatric field, due to its anatomical adaptability through size adjustments. Nonetheless, limitations related to somatic growth remain and require further investigation.

Leaflet #4 (GelMA-based) demonstrated superior cell viability (216.77 ± 77.69%) and scalability of the design through 3D bioprinting, positioning it as a promising prototype for the future development of heart valve tissue with growth potential. Consequently, the cytocompatibility evaluation was conducted exclusively for prototype 4, as it represents the most complete implant design and fulfilled the greatest number of predefined design criteria (Table S1). This prototype was selected based on its use of a biocompatible material, its scalability to pediatric dimensions via bioprinting, and its enhanced capacity to support cell viability and proliferation. Additionally, this study introduced the use of AoMAB cells for TEHV applications, highlighting their proliferative potential, as demonstrated by cytocompatibility tests in 3D cultures. Nevertheless, further studies are needed in this area, including additional in vitro validations and thrombogenicity assessments, to advance these approaches.

## Materials and Methods

### Materials

The list of materials is shown in Table S3.

### Prototypes and validation

Prototype designs were developed in consideration of 6 main design parameters:

1. Regeneration of valve tissue: Implants were constructed from biomaterials with favorable bioactivity and chemical stability.
2. Adaptation to child growth: An initial pediatric size applicable in clinical and in vitro cultures was selected, with a leaflet diameter of 10 mm, for which the scalable Ozaki model was used as a reference.
3. Safety of the valve implant: Antithrombogenic biomaterials with a limited risk of immunological rejection were utilized.
4. Mechanical properties: Biomaterials with properties similar to or superior to those of native tissue in terms of traction and elastic modulus were investigated.
5. Biocompatibility: Cell viability and proliferation in vitro were emphasized.
6. Local implant placement: Designs allowed for implant placement by transcatheter intervention.

On the basis of these parameters, 4 leaflet prototypes and one mitral valve implant prototype were manufactured via conventional manufacturing and bioprinting techniques. The selection of materials and the various comparisons made during their characterization are exclusively based on the design parameters.

#### Conventional manufacturing (leaflet prototype)

A 100-μm-thick UHMWPE membrane with dimensions of 1.56 mm × 1.95 mm × 1.90 mm was cut via a Trotec Laser S100 machine (GmbH, Austria). This UHMWPE membrane was tested and analyzed as leaflet #1. Leaflet #2 was prepared from UHMWPE leaflet #1 and coated with 5% PVA. The PVA solution was prepared by slowly adding 2 g of PVA to 40 ml of distilled water and maintaining the solution at 40 °C with agitation for 2 h to ensure complete dispersion and swelling. The solution was then stirred at 90 °C for 30 min to obtain homogeneity. The UHMWPE leaflet was immersed in the 5% PVA solution, frozen, and then freeze-dried for 24 h at a condenser temperature of −50 °C and a pressure of 1 mbar in a lyophilizer (LABCONCO, Fisher Scientific, USA). Leaflet 3 was prepared similarly but with a coating of 5% PVA mixed with 100 μg/ml collagen type I, followed by the same freezing and drying conditions as those used for leaflet #2. The samples for biological characterization were sterilized with ozone.

#### 3D printing

Leaflet #4 and the mitral valve implant prototype were fabricated using a bioink composed of 7.5% w/v GelMA (methacrylated gelatin) and 1 mg/ml LAP (lithium phenyl-2,4,6-trimethylbenzoylphosphinate) photoinitiator, utilizing volumetric bioprinting with the Readily3D Tomolite v2.0 (Lausanne, Suisse) Light doses of 180 mJ/cm^2^ were applied for crosslinking, with exposure times calculated at 25.7 s. The dimensions of the bioprinted leaflets matched those of the conventionally manufactured leaflet prototype (Fig. 1). The bioprinted implants measured 0.5 mm × 0.1 mm × 14.3 mm × 18.4 mm and were composed of 5% GelMA photoresin. Light doses of 350 mJ/cm^2^ and 400 mJ/cm^2^. The implants were subsequently preserved in phosphate-buffered saline (PBS) until characterization.

### Characterization of the leaflet prototypes

#### Microstructural study

The pore size of each leaflet material was measured from images obtained via a scanning electron microscope (FA-STE, SU5000, Hitachi, High-Technologies, Tokyo, Japan) via Hitachi map 3D software. Pore size was evaluated for 1 to 3 images of 50 pores per implant material via ImageJ v.1.54f bundled with 64-bit Java 8. Software (Wayne, Rasband, NIH, USA) was used. The equation used to calculate the pore size (Ps) was as follows (Eq. 1):

$$\mathrm{Ps}=1.5\times 2\times \frac{\sqrt{a^{2}+b^{2}}}{2}$$ (1)

Two techniques were employed to quantify the pores for each leaflet type via ImageJ and were applied to 3 leaflet samples of each material. The first followed a protocol established in previous literature [28], in which 50 pores per SEM image were manually selected as elliptical approximations. The ellipse areas were determined, and the major and minor axes were measured, from which the pore size was then calculated via the above formula.

The second technique sought to analyze pores by applying a threshold and particle detection algorithm in ImageJ (Wayne, Rasband, NIH, USA). Pores penetrating fully through the material were defined as regions on SEM images darker than a predefined, visually determined threshold. This threshold was kept the same for analysis of images of a given material and resulted in a binarized image of pore and nonpore regions. Images were then edited via the Analyze Particles plug-in. To encourage accurate counting, program-detected particles measuring under 100 μm were categorized as noise and therefore not included. Particles of all roundness values were considered. Details of this measurement technique are available in the Supplementary Materials (Table S2).

#### In vitro biodegradation

Enzymatic degradation was evaluated using 10-mm-diameter leaflet samples that were preweighed after being briefly wetted in PBS. Afterward, the samples were incubated at 37 °C with 0.1% collagenase type IV (>125 collagen digestion units/mg, Invitrogen) following a previously standardized concentration protocol. Degradation was assessed at 5 distinct time points: 1, 3, 7, and 14 d, with measurements performed in triplicate. Statistical analysis was conducted to evaluate the significance of the results across time points. Each time, 500 μl of 0.25 M EDTA was added to the relevant samples to stop the enzymatic reaction. Each implant was weighed on an analytical Sartorius BP211D (IG Instrument, Gesellschaft, Zürich, Switzerland), and the percentage of degradation was calculated relative to the initial sample weight.

#### Contact angle

Three samples of each leaflet were evaluated via an automated goniometer (Digidrop) and Digidrop software as well as a contact angle meter (https://gbxonline.com/) (GBX Etude des Technologies avancées) (GBX Scientific Ltd., Dublin, Ireland). This test was performed at room temperature by applying 4 drops (4 μl) of sterilized water on the surface of each leaflet sample.

#### Fluid sorption capacity

A gravimetric-based liquid sorption assay was conducted to assess the absorbance abilities as part of the overall physicochemical characterization of the leaflets. Leaflet samples were first left for 24 h in an oven at 60 °C to promote dehydration and then weighed on an analytical balance (Sartorius BP211D; IG Instrument, Gesellschaft, Zürich, Switzerland) (calibrated to within 0.1 to 0.01 mg accuracy). Afterward, 2-ml microtubes were prepared, each containing one leaflet sample and 2 ml of 0.1 M PBS (pH 7.4). The samples were stored in an incubator at 37 °C, and 3 replicates of each material at each time point were evaluated by measuring the final weight at 20-min intervals until 2 h after the addition of PBS. The excess water was removed by lightly dabbing the samples on filter paper prior to the final weight measurements. The fluid sorption capacity (FSC) was calculated via the following equation (Eq. 2):

$$\mathrm{FSC}\;\left(\%\right)=\left[\left(W_{1}–W_{0}\right)/\left(W_{0}\right)\right]\times 100$$ (2)

#### FTIR spectroscopy

Four samples of each leaflet prototype were evaluated via an FTIR spectrometer (Spectrum Two, Waltham, Massachusetts, USA). The spectra of the samples were read from 4,000 to 400 cm^−1^. This range was selected because it encompasses the principal functional groups of collagen (amide A, B, I, and II), UHMWPE (vinyl or acetyl groups), PVA (hydroxyl groups), and GelMA (methacrylic groups); each sample was subsequently scanned at a resolution of 4 cm^−1^/sample, with a PVA (1.0 mg) control sample used to confirm the chemical composition.

#### Simple mechanical strength test

The tensile modulus (dry) of each leaflet was obtained via a ZwickRowll universal testing machine (GmbH & Co. KG, Ulm, Germany) with 50-N capacity. The procedures followed the ISO 5840-1:2021 standards and ASTM D638 Type V [39]. A 50-N load cell calibration between 0.1 and 50 N was used, with a speed of 10 mm/min. Twelve dry samples per leaflet were tested at room temperature until rupture.

#### Dynamic mechanical analysis

Leaflet samples were further analyzed using DMA to assess material properties under dynamic conditions and identify variations in viscoelastic parameters. The prototype thicknesses were approximately 0.8 and 2 mm for the UHMWPE-based samples and GelMA, respectively. Each sample was sequentially mounted on the DMA 242 E Artemis (NETZSCH) instrument to undergo compression testing. The furnace chamber temperature was set to 37 °C after a heat ramp rate of 2 °C/min and 20 min allotted for stabilization. The temperature during testing ranged from approximately 36 to 39 °C. The force track was 120% with an oscillating stress at several different frequencies—0.5, 1, 2, and 10 Hz—to determine the sample elastic storage modulus (*E*′), loss modulus (*E*″), and damping factor (tan δ) for each material. The data were analyzed at a constant frequency of 1 Hz, and graphs were created with NETZSCH Proteus software.

#### Nanostructural characterization

Dry leaflet samples were evaluated via atomic force microscopy (AFM) quantitative nanomechanical mapping (QNM) Bruker dimension Econ with ScanAsyst (Massachusetts, USA). Tests were conducted by tapping (contact mode) at room temperature (21 °C) a ScanAsyst at resolution of 512 × 512 data points at 1 Hz via an SNL-10 tip and XE-100 model from Park System. The nanotopography, elastic modulus, adhesion, and stiffness were measured with spring constant of 0.58 nN/nm. The obtained images were analyzed via Gwyddion v2.65 software (http://gwyddion.net/download.php).

#### Cytocompatibility of the leaflet prototype

AoMABs were isolated and expanded to passage 11 via a protocol described previously [8].

T25 was flask coated with 1% collagen type I (Sigma-Aldrich). The medium used for AoMAB proliferation was prepared with 82% IMDM GlutaMAX (Gibco, 31980-030), 15% heat-inactivated fetal bovine serum (A5256701), 1% penicillin/streptomycin, insulin–transferrin–selenium (100×), 1% nonessential amino acids (100×), (100 μl) 2-mercaptoethanol (100 mM), and (5 μl) human basic fibroblast growth factor (0.1 μg/ml). The cultures were maintained at 5,000 cells per cm^2^ in a humidified incubator with 5% CO_2_ and were split every 2 to 3 d.

##### Viability test

AoMAB cells were seeded on 2D transparent 96-well plates (confluent after 1 d), the media were removed, and 50 μl of the mixture in each well was supplemented with Calcein-AM (Thermo Fisher, #C1430) at 1 mg/ml (5 μl), propidium iodide (Fluka, 81845) at 1 mg/ml (5 μl), Hoechst (1×) at 0.4 μl, and Dulbecco’s PBS (Sigma, D8537) at 1 ml. Then, the plate was incubated for 20 min at 37 °C and 5% CO_2_, and the number of cells with an automated cell counter (RWD, USA) was determined. 2D AoMAB cell culture was used as a positive control.

##### Cell proliferation

Four samples of each leaflet #1 to #4 prototype were sterilized with ozone SteriLux (Switzerland) for at least 15 d before the experiment. AoMAB cells (10^6^) were seeded on each leaflet (10 mm in diameter) in 1 ml of proliferation medium and subsequently incubated (37 °C, 5% CO_2_) for 2, 5, and 10 d. Each time the medium was removed from the well and washed with 1× PBS, the leaflet was incubated with 500 μl of proliferation medium and 500 μl of CyQUANT direct cell proliferation mixture for 1 h at 37 °C; then, 200 μl was transferred to a 96-well plate, and the fluorescence was measured at a 508-nm peak excitation wavelength. The absorbance was obtained with a FlexStation 3 spectrophotometer (Molecular Devices; California, USA) and interpolated into a calibration curve of AoMAB cells.

##### Cell adhesion

CellTracker Green CMFDA C7025 (50 μg) was used to adherent cells according to the manufacturer’s protocol. First, the culture medium was removed, the prewarmed CellTracker working solution (10 μM) was added, and the samples were incubated for 40 min (37 °C, 5% CO_2_). Then, the CellTracker was removed, and the proliferation medium AoMAB was added. Finally, images were obtained with an Axio Observer fluorescence microscope (ZEISS, Germany).

#### Cytocompatibility of the Valcard prototype of mitral valve implant

A 3D-printed prototype made of 5% GelMA preserved in 1× PBS was seeded with AoMAB (1 × 10^6^ cells/ml in 5 ml) in proliferation medium 7 d after bioprinting and cultured for 14 d, after which the medium was changed every 2 d. Finally, the 3D cultures were fixed with 4% paraformaldehyde (PFA) for 30 min. Samples previously fixed were washed with PBS, and after adding 0.1% PBS–sodium azide, they were preserved at 4 °C for immunofluorescence.

Immunostaining analysis: The fixed samples were permeabilized with 0.1% Triton in 1× PBS for 30 min, washed with 1× PBS on a shaker 3 times for 5 min each, and then blocked with 1% PBS–BSA (bovine serum albumin) for 10 min at room temperature. The primary antibody mouse anti-vimentin (1:1,000, ab20346, Abcam, Cambridge, MA, USA) was subsequently added to the samples, which were subsequently incubated overnight at 4 °C. The secondary antibody Alexa 488-conjugated anti-mouse (1:500, A-11001, Invitrogen, Waltham, MA, USA) was subsequently added for 1 h at 37 °C. The samples were washed with 1% BSA and 0.1% BSA for 5 min each before being washed twice in PBS for 5 min. The nuclei were stained with 4′,6-diamidino-2-phenylindole (DAPI) (1:1,000, Sigma-Aldrich, 022M4004V, GmbH, München, Deutschland), and the cytoskeleton was stained with phalloidin-532. Images were acquired with a Nikon eclipse Ti2 confocal microscope (Nikon, Tokyo, Japan) with dimensions of 10× and 40×, Plan Apo objectives LWD (Nikon; Tokyo, Japan) software Nis-Elements AR 5.41.02, SPED/COLM light-sheet microscope, and MesoSPIM Olympus MVPLAPO 1 X (Olympus, Tokyo, Japan).

### Statistical analysis

R Project statistical analysis software (R Foundation for Statistical Computing, Vienna, Austria, R version 4.0.2, 2020) and GraphPad Prism 8 were utilized (GraphPad, USA) for the analysis. The significant differences are reported at *P* < 0.05 according to the analysis of variance (ANOVA) results (1- and 2-way ANOVA).

## Data Availability

The data that support the findings of this project are available from the corresponding author upon reasonable request.
