# Supplementary material for: Tissue Engineering In Vitro Leaflet- and 3-Dimensional Printing-Based Implant Prototypes for Infant Mitral Valve
Source: BME Front. 2025 Aug 7;6:0159. doi: 10.34133/bmef.0159 (PMC12329791; doi:10.34133/bmef.0159)
Supplement: Supplementary 1 — Figs. S1 to S6 Tables S1 to S3 Movies S1 to S3 [file bmef.0159.f1.docx]

Title

Tissue Engineering in vitro leaflet and 3D printing-based implant prototypes for infant mitral valve.

**Authors**

Martha I. González-Duque^1,2,3*^, Arielle Breuninger^1^, Frédéric Leis^4^, Julio B. Michaud^1^, Shaginth Sivakumar^1^, Vincent Pautu^5^, Marisa E. Jaconi^4^, Marc Jobin^5^ and Adrien Roux^1^*

**Affiliations**

^1^^.^ Tissue Engineering Laboratory, Bioengineering Group, HEPIA HES-SO University of Applied Sciences and Arts Western Switzerland, Geneva, Switzerland.

^2.^ Tissue Engineering Group, Departamento de Farmacia, Facultad de Ciencias, Universidad Nacional de Colombia, Av. Carrera 30 # 45-10, Bogotá 111321, D.C., Colombia.

^3.^ Biomedical Sciences Group, Department of Medicine, Universidad Antonio Nariño. Carrera 1 #47a – 15, Bogotá D.C., Colombia.

^4.^ Department of Basic Neurosciences, Faculty of Medicine, University of Geneva, Switzerland.

^5.^ Materials, Optics and Nanotechnology Group, HEPIA HES-SO University of Applied Sciences and Arts Western Switzerland, Geneva, Switzerland.

*Address correspondence to: Adrien Roux; [adrien.roux@hesge.ch](mailto:adrien.roux@hesge.ch)

**Abstract**

**Objective:** This study engineers leaflet- and 3D printing-based implant prototypes for infant mitral valve repair via *in vitro* cultured mesoangioblasts isolated from the human fetal aorta (AoMAB).

**Impact Statement:** Ultrahigh-molecular-weight polyethylene (UHMWPE) coatings, as well as 3D-printed gelatin methacrylate (GelMA) hydrogels for implants, represent new possibilities for devices used in mitral valve repair.

**Introduction:** Mitral valve prolapse (MVP) repair in pediatric patients is challenging due to somatic growth, patient–prosthesis mismatch, reinterventions, infections, and thromboembolism. Tissue-engineered heart valves (TEHVs) offer potential solutions through conventional and 3D printing biofabrication.

**Methods:** Four materials are evaluated: ultrahigh-molecular-weight polyethylene (UHMWPE), UHMWPE coated with polyvinyl alcohol (PVA), UHMWPE coated with PVA and collagen, and 3D-printed gelatin methacrylate (GelMA) hydrogels. The prototypes are characterized for micro/nanostructural, physicochemical (degradation, contact angle, Fourier transform infrared spectroscopy), and mechanical properties (simple strength tests, dynamic mechanical analysis) and assessed for cytocompatibility using AoMAB cells. A 3D-printing mitral valve prototype is analyzed via immunostaining.

**Results:** highlight UHMWPE coated with PVA and collagen as the most promising, with degradation (7.30% ± 18.71), a hydrophilic contact angle (26.13° ± 1.45), and biocompatibility (177.04% ± 68.92% viability). GelMA prototypes show superior viability (216.77% ± 77.69%) and scalability for 3D printing.

**Conclusion:** UHMWPE coated with PVA and collagen and GelMA demonstrate strong potential for TEHVs, with AoMAB cells facilitating 3D culture and future personalized pediatric applications. Further *in vitro* validation and thrombogenicity assessments are needed.

**Keywords**

3D printing; infant; implant; leaflet; mitral valve; tissue engineering.

**INTRODUCTION**

Mitral valve prolapse (MVP) is a common congenital valve anomaly and the most frequent indication for mitral valve surgery [1]. The gold standard treatment for mitral valve disease is mitral valve replacement with an implant; however, it poses a significant challenge for pediatric surgical intervention due to the fragile, immature, and small nature of the mitral valve mainly in neonates [2]. Additionally, there are difficulties in obtaining prostheses of appropriate sizes. Pediatric patients must use anticoagulants for extended periods, and their long-term outcomes, as well as the high risks associated with repeated surgical interventions, are less favorable compared to the adult population [1].

Despite continuous improvements of surgical therapeutic approaches with mechanical or bioprosthetic implants, the mortality rate remains high: approximately 17.9% -28.6% of patients do not survive seven years postsurgery [3]. These unsuccessful cases often stem from complications such as thromboembolism, immune rejection, calcification, implant degradation, and infection [4].

Consequently, the main challenges in improved valve implant design for infants are related to maintaining synchronous growth of the implant, size, resistance to infections or thrombosis, and as well as durability to avoid surgical reintervention, advancements in materials with antithrombogenic coatings, and the design of small-scale drug delivery systems for transcatheter valves, tailored to patient-specific needs [5]. This therapeutic approach relies on personalized designs that are anatomically adapted to the patient to avoid patient–prosthesis mismatch (PPM) associated with somatic growth. The issue arises from the limited of tissue repair-focused therapeutic alternatives, that are specifically designed to address the unique needs of pediatric patients.

Tissue engineered heart valves (TEHVs) seek to mimic native mitral valve tissue and address tissue needs through their three-dimensional structure, in which each design parameter corresponds to a specific physiological need. This objective is achieved by selectively incorporating cells, biomaterials, and bioactive factors while considering biomaterial characteristics, valve development techniques, cell origin bioactive factors involved in cell differentiation, and the mechanical properties of the implant due to crosslinking and other processes. Specifically, previous studies have proposed applying valvular interstitial cells (VICs) and valvular endothelial cells (VECs) directly onto a scaffold to promote proliferation and the formation of native tissue ECM. Other studies have utilized induced pluripotent stem cells (iPSCs) and genome editing techniques [6].

Isolating primary human VECs and VICs is challenging, and their proliferative capacity is limited. Therefore, exploring alternative cell types for bioprinting is essential. One such promising candidate for cardiovascular applications and use in tissue-engineered heart valves (TEHVs) is mesoangioblasts isolated from the human fetal aorta (AoMABs) [7]. These cells are characterized as a subpopulation of pericytes or vessel‐associated stem/progenitor cells capable of self‐renewal and differentiation into various mesoderm cell types, including skeletal and cardiac muscle. The high proliferation rate and differentiation capabilities of AoMAB cells, along with their expression of common mesenchymal phenotypic markers shared with VICs, as well as their ability to differentiate into osteogenic, myofibroblastic, and chondrogenic lineages, make them well-suited for *in vitro* studies of cellularized heart valve implants [7].

The scaffold manufacturing process is also a key component of valve implant design. Tissue engineering via 3D bioprinting allows cellular hydrogel constructs to be made with patient- specific anatomical geometry and mechanical properties with customized printability, shape fidelity and bioactivity [8]. In the pediatric field, advancements in implant size, the use of autologous or allogenic cells, and the implementation of personalized designs enhance biocompatibility, reduce mismatches, and incorporate high-quality control measures to facilitate scalability with limited environmental impact [9]. However, the disadvantages are primarily associated with the high costs of bioprinting processes today, as well as the maneuverability of the delivery systems.

The aim of this study was to engineer and characterize leaflet- and 3D printing-based implant prototypes for infant mitral valve repair using *in vitro* cultured AoMAB cells.

Consequently, the selection of materials for the leaflet and implant prototype is of paramount importance in both the bioprinting process and conventional manufacturing. Some of the most prominent materials in use for constructing TEHV scaffolds are synthetic biopolymers and naturally occurring materials. In the case of the former, popular polymers include polyglycolic acid, poly(lactic–co– glycol) (PLGA) and polyhydroxyalkanoates, whereas alternatives such as collagen, elastin, alginate, cellulose, gelatin, hyaluronic acid, chitosan and keratin are widely used materials for the latter [10].

For instance, previous studies have investigated the volumetric bioprinting properties of gelatin methacrylate (GelMA), a biocompatible and osmotic material derived from porcine gelatin, which exhibits optimal degradation time, cytocompatibility, and supports cell migration [11]. Methacrylate groups incorporated into its structure enable crosslinking with a photoinitiator under ultraviolet light exposure, thereby enhancing its structural stability while preserving a consistent refractive index during polymerization [12]. Similarly, polyvinyl alcohol (PVA) is a synthetic polymer with high clinical promise in TEHV applications because it is highly compatible with both a hydrogel and a freeze-dried material, is characterized by physical cross-linking, and is hydrophilic. Given that hydrophilicity has been linked to biocompatibility, blending PVA with other molecules has been shown to improve cell adhesion and growth *in vitro* [13]. Collagen is also an encouraging material due to its biocompatibility, low immunogenicity, and potential for controlled and customizable biodegradation [14].

Nonetheless, one of the most compelling materials currently being explored for TEHVs is ultrahigh-molecular-weight polyethylene (UHMWPE). A synthetic polymer, UHMWPE, possesses a uniquely favorable balance between a low coefficient of friction and high wear and fatigue resistance [15]. Furthermore, the material is flexible, antithrombogenic, antibacterial, and biocompatible in cardiovascular and orthopedic applications [16]. Several limitations of UHMWPE particles exist—including relatively low thermal stability and low load-bearing capacity—although incorporating UHMWPE-based micro- and nanocomposites could be an effective solution for addressing these mechanical shortcomings [15].

These implant base materials can be implemented in the final design through a variety of methods, including laser cutting of desired shapes or through 3D printing techniques. Specifically, recent studies have described the successful use of 3D bioprinting via extrusion, laser, robotic arm, and acoustic techniques or through volumetric additive manufacturing with photosensitive resin [17].

The design possibilities in bioprinting vary greatly from standard mass-produced prototypes to patient-specific implants. However, standard bioprinting procedures could be effectively applied in cases such as printing of Ozaki templates for aortic valve reconstruction, where autologous pericardium obtained surgically must have defined, measured values cut for implantation [18].

This study explores leaflet- and 3D printing-based prototypes using UHMWPE, UHMWPE-PVA, UHMWPE-PVA + collagen, and GelMA hydrogels. These materials were evaluated for their structural, physicochemical, mechanical, and cytocompatibility properties with AoMAB cells. A 3D-printed valve prototype was also analyzed through immunostaining to assess biological integration.

**RESULTS**

**Prototypes and Validation**

We generated four leaflet prototypes with the same dimensions (**Fig. 1**). Leaflet #1 (control) was made of 100 µm UHMWPE; leaflets #2 and #3 were developed via conventional coating and freeze-drying techniques using UHMWPE coated with PVA and PVA + collagen, respectively. Leaflet #4 was constructed from 3D-printed GelMA. For all the scaffolds, the leaflets were 10 mm in diameter and adhered to the Ozaki model for leaflet- based implant design [19,20], with modifications for *in vitro* culture in 24- and 48- well plates. The mitral valve prototype model—ValCard—was 3D printed for postprinting culture on 12-well plates. This 3D culture design was validated through established design parameters based on material biocompatibility and mechanical properties (**Table S1**).

**
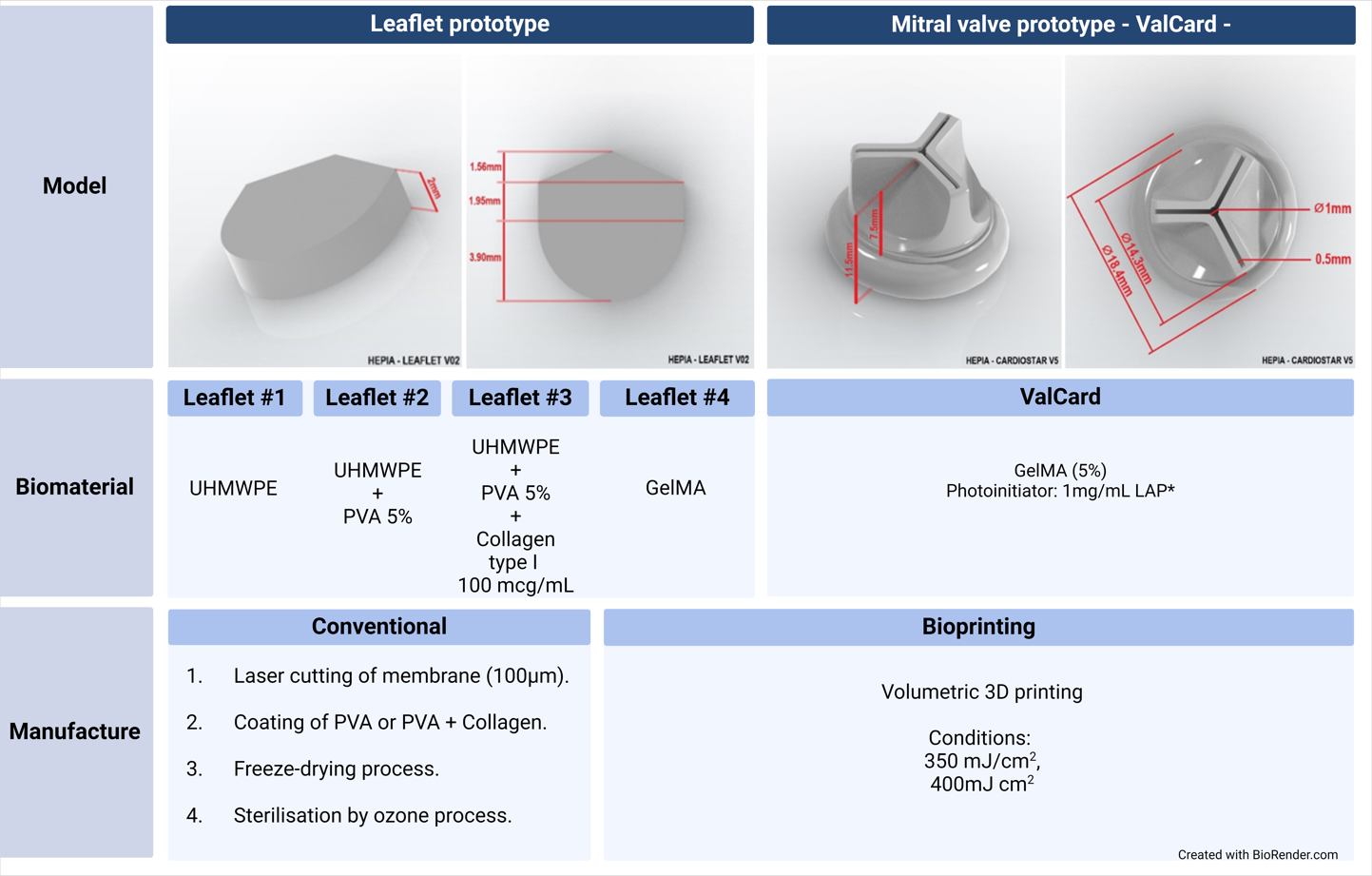
 Fig. 1.** Design and biomaterials for leaflets and mitral valve prototypes.

**Characterization of the leaflet prototypes**

We characterized the four leaflet prototypes according to the following properties: microstructure, physicochemistry, mechanics, nanostructure, and cytocompatibility. Valcard, the newly engineered mitral valve prototype proposed in this study for implant application, was evaluated with AoMAB cells in culture.

*Microstructural study*

Microstructural characterization results obtained from surface of leaflets #1--#4 via environmental scanning electron microscopy (ESEM) are presented in the microstructural characterization in **Fig. 2**. Leaflet #1 exhibited ill-defined surface pores and UHMWPE fibers; in leaflets #2 and #3, well-defined pores with structured multiaxial fibers were observed. Given the hydrogel structure of GelMA for leaflet #4, these samples were subjected to a freeze‒drying process for observation via ESEM, and analysis demonstrated evidence of well-defined pores as well as laminar edges without interconnection on the surface.

**
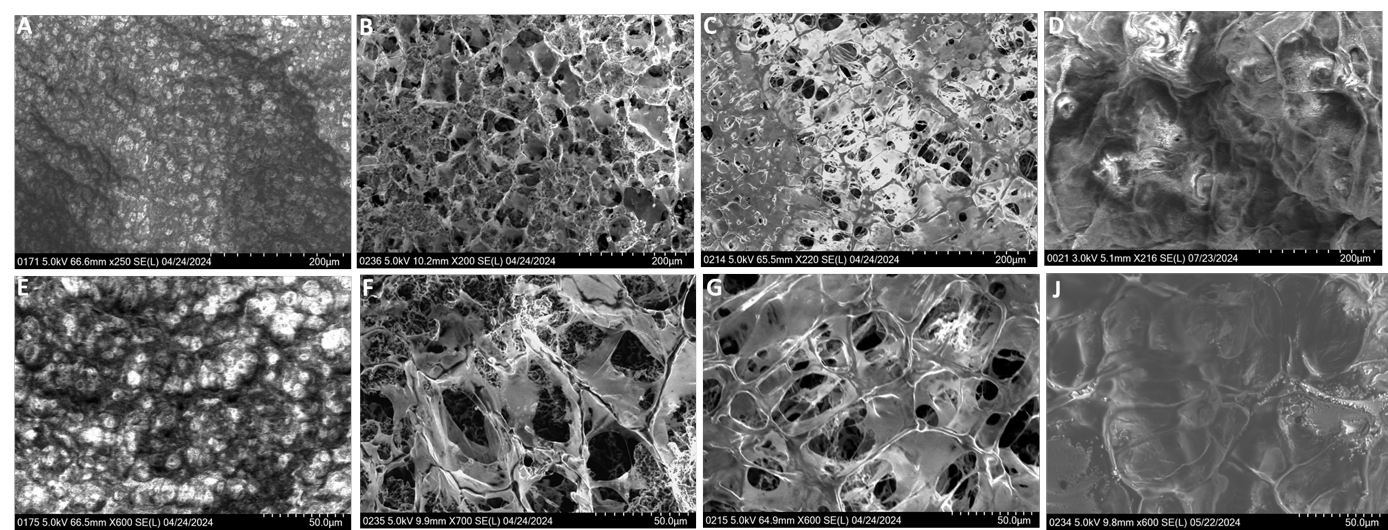
 Fig. 2.** ESEM images of the surface of the leaflet prototypes. (A, E) Leaflet #1 UHMWPE; (B, F) Leaflet #2 UHMWPE+PVA 5%; (C, G) Leaflet #3 UHMWPE+PVA 5%+100 µg/mL collagen; (D, H) Leaflet (4) GelMA 7.5% w/v. Scale bars: 200 µm (A-D) and 50 µm (E-J).

**Table 1** reports the pore size was determined via two different techniques with ImageJ software. The pore area results calculated via Technique 1, which relies on a manual selection process, closely align with the trends demonstrated by the pore diameter results. Furthermore, there was statistically significant difference (p ≤ 0.05) in pore diameter was detected between leaflets #1 (11.26 µm ± 2.60) and leaflets #2 (50.37 µm ± 18.96) and #3 (44.92 µm ± 17.18). Similarly, leaflet #4 (17.82 µm ± 5.84) was significantly different from leaflets #2 and #3.

| Characteristic of leaflet | #1 UHMWPE | #2 UHMWPE + PVA | #3 UHMWPE + PVA + COLLAGEN TYPE I | #4 GelMA |
| --- | --- | --- | --- | --- |
| Pore size (area). | 10.30 ± 4.82 | 228.32 ± 182.39 | 185.91 ± 148.38 | 29.55 ± 22.94 |
| Pore size (area). Technique 2 (µm^2^) | 16.44 ± 27.15 | 28.64 ± 52.21 | 22.34 ± 43.07 | 24.71 ± 23.78 |
| Pore size (diameter) Technique 1 (µm) | 11.26 ± 2.60 | 50.37 ± 18.96 | 44.92 ± 17.18 | 17.82 ± 5.84 |

**Table 1.** Pore size of the leaflet prototypes. The values are presented as the means ± SDs: p ≤ 0.05: pore size (area) Technique 1: (#1-#2; #1-#3; #2-#4; #3-#4) n=50 pores. p ≤ 0.05: pore size (area). Technique 2: (#1-#2; #2-#3) n= (#1=575 pores; #2= 2554 pores; #3= 1206 pores; #4=12 pores). p ≤ 0.05: pore size (diameter) Technique 1: (#1-#2; #1- #3; #2- #4; #3-#4) n=50 pores.

The pore sizes of leaflets #2 and #3 were indicative of macropores; however, while the effective pore size obtained in these prototypes was found to be less than 100 µm, this value was much greater than the average pore size obtained from the uncoated UHMWPE particles (leaflet #1). Technique 2 calculates the pore size (area only) via a threshold-based automatic detection program capable of detecting pores of a far greater size range, specifically very small pores present in the material. Consequently, these results revealed high standard deviations in pore size for each leaflet, with statistical significance (p≤0.05) observed only between leaflets #1 (16.44 ± 27.15) and #2 (28.64 ± 52.21), as well as between leaflets #2 (28.64 ± 52.21) and #3 (22.34 ± 43.07). Further details on the results and pore classification are reported in the **Table S2.**

*In vitro biodegradation*

The percentage of degradation by collagenase IV was determined at 6 different time points over the course of 14 days. Overall degradation by day 14 was measured for leaflets #1 (7.87% ± 17.14), #2 (37.31% ± 9.66), and #3 (7.30% ± 18.71). Leaflet #4 was not evaluated due to structural limitations in conducting the assay on a hydrogel. Polymethylmethacrylate (PMMA) was utilized as a negative control sample (-0.02% ± 1.19 at 14 days). As outlined in **Fig. 3a,** significant differences (p ≤ 0.05) were observed on day 14 between the following prototypes: leaflets #2 and PMMA, leaflets #3 and PMMA, leaflets #1 and #3, and leaflets #1 and #2. Furthermore, the addition of collagen to a PVA-only leaflet coating resulted in an overall lower level of leaflet degradation. Values less than 0 were reported as equal to 0; specific test data are recorded in **Fig. S2-S3.**

*Contact angle*

Leaflet hydrophilicity was quantified via contact angle measurements with deionized water, as shown in **Fig. 3b**. Leaflet #1 presented a far greater contact angle than the other leaflet samples did, with a value around the hydrophilicity limit (91.90° ± 1.57). The UHMWPE- based leaflets with coatings exhibited significantly smaller contact angles, at 23.74° ± 5.57 for leaflet 2 and 26.13° ± 1.45 for leaflet #3. Leaflet #4 also had a relatively low contact angle (31.90° ± 11.70), and these latter three materials were all found to be hydrophilic. Significant differences (p ≤ 0.05) between leaflets #1 and leaflets #2, #3 and #4 were detected.

*Fluid sorption capacity*

The fluid sorption capacity of each leaflet was evaluated to simulate implant interactions with native tissue in aqueous media (**Fig. 3c**). The samples were analyzed over the course of 2 hours (with measurements every 20 minutes). Values less than 0 were approximated to 0 on the graph (Figure S4). At 120 min, leaflet #3 had the greatest percentage change in weight due to sorption (91% ± 93.81) and was significantly different (p ≤ 0.05) from all the other leaflets and the controls. The average fluid sorption capacity for leaflet #1 (3.70% ± 6.41), leaflet #2 (5.55% ± 33.67) and the negative controls polymethyl methacrylate (PMMA) (2.70% ± 0.48) and polystyrene (PS) (1.19% ± 0.59) generally displayed negligible sorption values (See **Fig. S4-S5**).

*
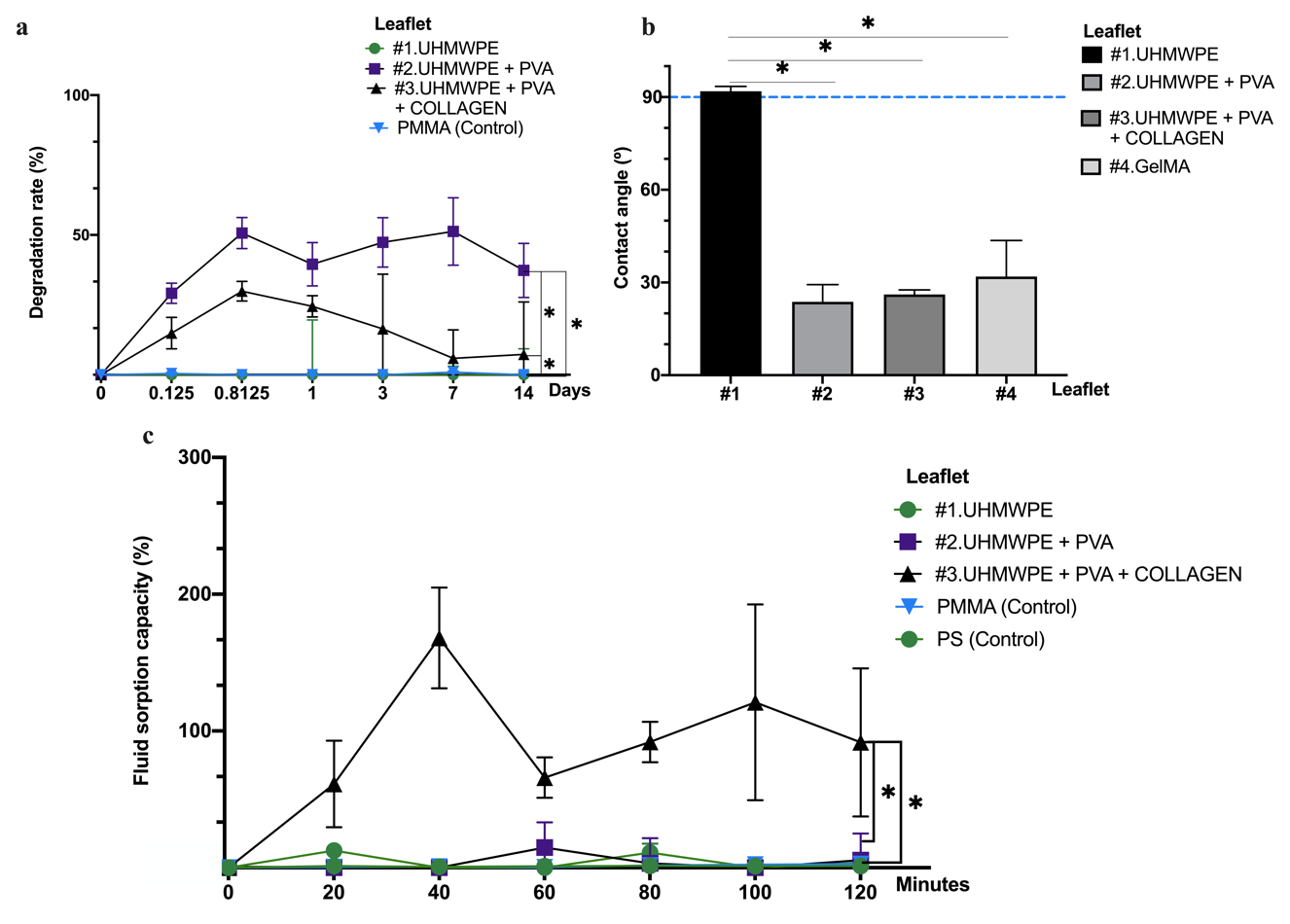
*

**Fig. 3.** Assessment of degradation rate, contact angle and fluid sorption capacities of Leaflet #1 UHMWPE; Leaflet #2 UHMWPE+PVA 5%; Leaflet #3 UHMWPE+PVA 5%+100 µg/mL collagen type I; Leaflet #4 GelMA 7.5% w/v (a) Degradation rate for each leaflet prototype (%). Control: polymethyl methacrylate (PMMA). n= 3; mean ± SD. Significant differences: *p ≤ 0.05. (b) Contact angle (°), n= 4; mean ± SD. Significant differences: *p ≤ 0.05. (c) Fluid sorption capacity (%) percentage. Control: polymethyl methacrylate (PMMA) and polystyrene (PS). n= 3; mean ± SD. Significant differences: *p ≤ 0.05.

*Fourier transform infrared (FTIR) spectroscopy*

We determined the chemical composition of each leaflet prototype using FTIR analysis as depicted in **Fig. 4**. The leaf #1 spectra presented characteristic peaks at 2914 cm^-1^ and 2847 cm^-1^ (products of polyethylene oxidation as well as hydrogen bonded and nonbonded hydroperoxide), 1462 cm^-1^ (vibrations for ethers and other groups), and 719 cm^-1^ (absorption for transethylene groups) (see **Fig. 4a**). Leaflet #2 exhibited spectra consistent with those of UHMWPE and PVA, with characteristic peaks at 834 cm^-1^ (C-C), 1087 cm^-1^ (C-O)-C-OH), 1318 cm^-1^ (-C-O-C), and 3260 cm^-1^ (OH stretching) (see **Fig. 4b**). Leaflet #3 spectra presented peaks characteristic of UHMWPE, PVA, and collagen type I at 3247 cm^- 1^ (amide A), 2905 cm^-1^ (amide B), 1628 cm^-1^ (amide I), and 1531 cm^-1^ (amide II) (see **Fig. 3c**). Leaflet #4 displayed absorption spectra expected for GelMA, with peaks at approximately 3346 cm^-1^ (O-H and N-H stretching) between 2800 cm^-1^ and 3100 cm^-1^ (C- H groups), 1631 cm^-1^ (amide I), and 1531 cm^-1^ (amide II) (see **Fig. 4d**). PVA was used as a positive control (see **Fig. 4e**) and exhibited typical absorption peaks at approximately 834 cm^-1^ (C-C), 1077 cm^-1^ ((C-O)-C-OH), 1318 cm^-1^ (-C-O-C), and 3260 cm^-1^ (OH group stretching). **Fig. 4f** shows all the leaflet prototypes (#1-#4), as well as the PVA, which presented typical absorption peaks according to their respective chemical compositions.


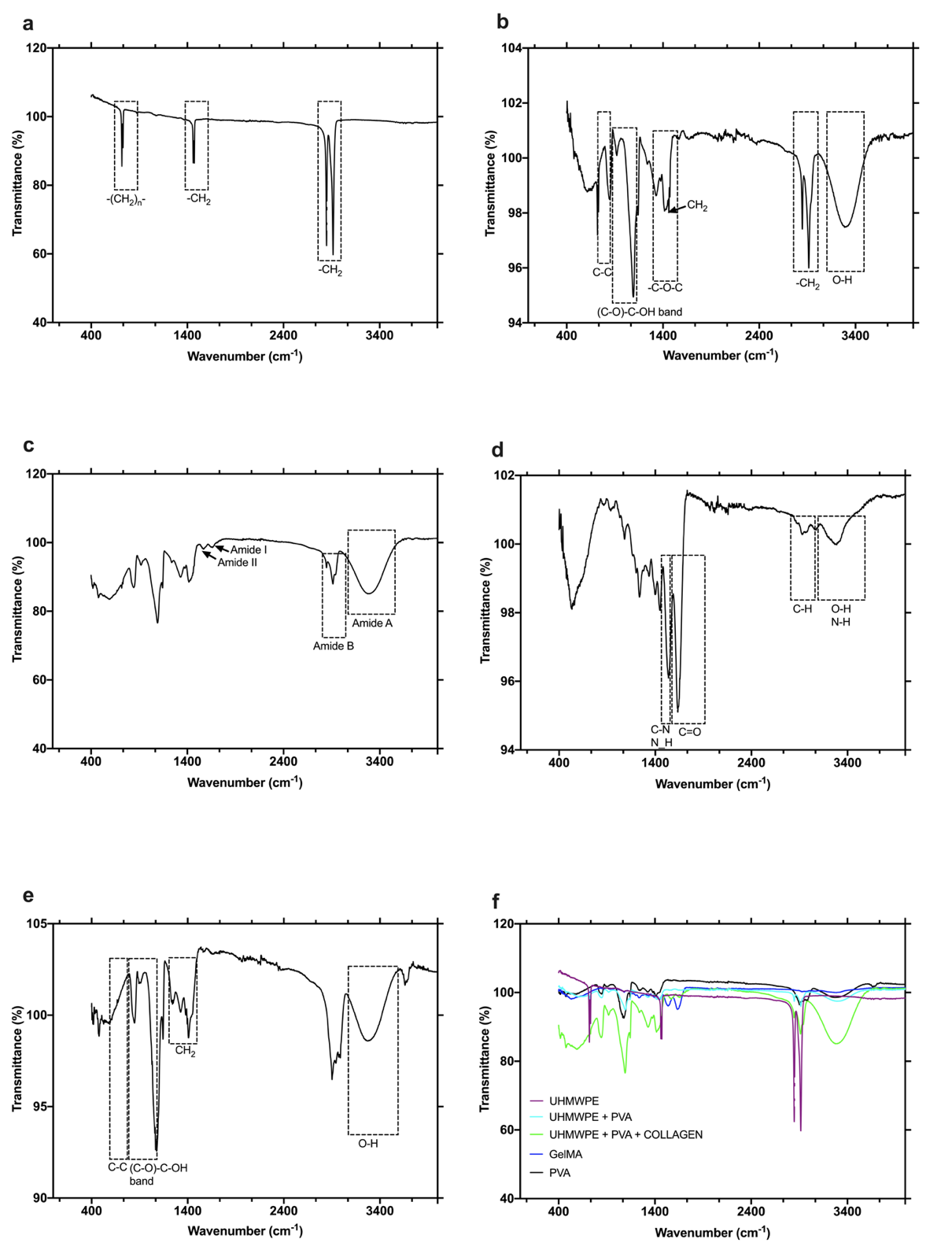


**Fig. 4.** FTIR analysis of the leaflet prototypes. n=4; (a) Leaflet #1 UHMWPE; (b) Leaflet #2 UHMWPE+PVA 5%; (c) Leaflet #3 UHMWPE + PVA 5% +100 µg/mL collagen type I; (d) GelMA 7.5% w/v. (e) Control (PVA); (f) Spectra of leaflets #1--#4 and the control.

*Simple mechanical strength test*

We performed tensile strength mechanical testing was performed for each leaflet to assess prototype strength and elasticity. We found that Neither the PVA nor the PVA + collagen type I coatings provide a statistically significant (p ≥ 0.05) effect on the maximum force, tensile strain, elastic modulus, force break, or force at 0.2% plastic strain for the UHMWPE- based leaflets (see **Table 2**). The elastic modulus decreased slightly for leaflet #2, likely was attributed to the PVA coating.

**Table 2.** Simple mechanical strength test

| **Mechanical properties** | **#1** **UHMWPE** | **#2** **UHMWPE + PVA** | **#3** **UHMWPE + PVA + COLLAGEN TYPE I** |
| --- | --- | --- | --- |
| Maximum force (N) | 3.622 ± 0.105 | 3.612 ± 0.124 | 3.582 ± 0.268 |
| Tensile strain (displacement) at break (standard) (%) | 9.175 ± 0.884 | 9.45 ± 1.623 | 9.041 ± 1.850 |
| Elastic modulus (GPa) | 0.190 ± 0.021 | 0.177 ± 0.025 | 0.179 ± 0.022 |
| Force Break (N) | 3.601 ± 0.111 | 3.59 ± 0.121 | 3.557 ± 0.271 |
| Force at 0.2% plastic strain (N) | 0.907 ± 0.154 | 0.850 ± 0.166 | 0.842 ± 0.139 |

The values are presented as the means ± SDs: p ≥ 0.05 (ns) indicates no significant differences in mechanical properties between leaflets #1, #2 and #3.

*Dynamic mechanical analysis (DMA)*

We performed dynamic mechanical analysis on leaflets #1-#4 (**Fig. S6**) in the temperature range of 37.5–39.5°C, and assessed the storage modulus (E’), loss modulus (E”), and loss factor (tan δ). For each leaflet, the initial E’ was found to be approximately 2–35 MPa, whereas the initial E” was approximately 2-6MPa. Furthermore, the E’ curves for leaflets #2 and #3 exhibited a stability in this temperature range. The curve of tan δ was indicative a specific shape that corresponded to the energy transitions among the layers of these composite materials for leaflets #2 and #3. Leaflet #4, however, exhibited an upward E’ curve with a peak at 35 MPa and generally displayed mechanical behavior distinct from those of the other three thermoplastic leaflets. As UHMWPE-based leaflets, their respective values for E’ were instead relatively low and stable.

*Nanostructural characterization*

Additionally, we evaluated the nanostructural properties of the leaflets (**Fig. S7a-d**). The force maps revealed differences among the four different leaflets, with leaflet #1 being more homogeneous than leaflets #3 and #4. Leaflet #1 demonstrated a topography typical of UHMWPE—given the presence of elevations on its surface in a structured manner—while elevations in the topography of leaflet #2 were largely irregular. The surface of leaflet #3 was regularly patterned, although with a defined rough appearance. Finally, leaflet #4 exhibited a smooth nanoscale topography with a concave curvature that was generally void of major elevations (**Fig. S7e-h**). The nanoscale mechanical properties varied significantly among the prototypes, with leaflet #4 possessing the lowest elastic modulus, and the biomaterial coatings greatly reduced the elastic modulus of the UHMWPE-based leaflets. Variations in adhesion and stiffness were also observed in this characterization, with values once again resting notably lower for leaflet #4.

*Cytocompatibility of the leaflet prototype*

*Viability test:* We evaluated the viability of AoMAB cells for each leaflet to be above 70% among all prototypes (see **Fig. 5a**), a parameter that highlights their respective compatibility with these cells. The highest viability was obtained by leaflet #4 (GelMA), at 216.77% ± 77.69%, followed by leaflet #3, at 177.04% ± 68.92%. Leaflets #2 and #1 presented viabilities of 114.33% ± 38.05% and 87.79% ± 28.11%, respectively. No significant differences were found among the prototypes.

*Cell proliferation:* We assessed the cell proliferation chart for each leaflet (**Fig. 5b**), with AoMAB cells in conventional 2D culture as a control. The samples were evaluated at three different time points (0, 2 and 10 days). Maximum proliferation was generally obtained at 10 days postseeding. Leaflet #2 presented the greatest variability in average cell count as a function of time, whereas the most stable prototype over time was leaflet #3.

*Cell adhesion:* Adhesion testing was used to quantify the rate of cell adhesion to the leaflet scaffolds 5 days after seeding. **Fig. 5c** displays the different cell adhesion patterns associated with each type of leaflet. While leaflets #1 and #2 exhibited a superficial pattern of adhesion, leaflet #3 demonstrated greater adhesion to the surface of the material, and leaflet #4 generated a concentric pattern. These patterns can be attributed to cell proliferation trends induced by a specific leaflet biomaterial. Fluorescence intensity quantification for each leaflet is presented in **Fig. S8.**


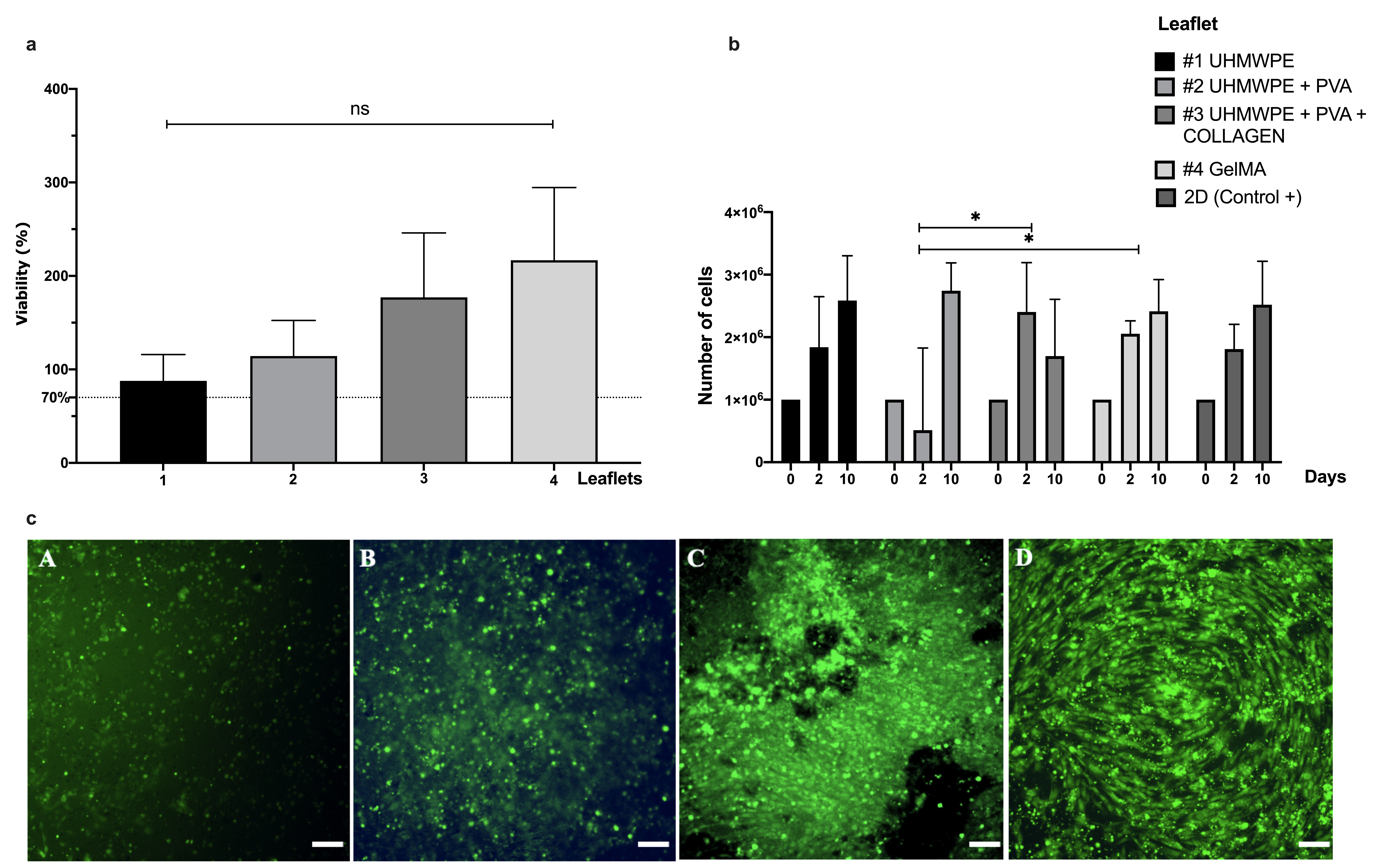


**Fig. 5.** Cytocompatibility of AoMAB cells within leaflets. (a). Cell viability percentage (%) n=3 mean ± SEM: p ≥ 0.05 (ns) show no significant difference. Leaflet #1 UHMWPE; Leaflet #2 UHMWPE + PVA; Leaflet #3 UHMWPE + PVA + Collagen type I; Leaflet #4 GelMA. (b) AoMAB proliferation on leaflets. n=3 mean ± SD: p ≥ 0.05 (ns) indicates no significant differences. Leaflet #1 UHMWPE; leaflet #2 UHMWPE + PVA; leaflet #3 UHMWPE + PVA + Collagen type I; leaflet #4 GelMA; 2D AoMAB cells culture were used as a positive control (+). Time (0, 2, 10, days). (c) Cell adhesion over leaflets (5 days). (A) Leaflet #1 UHMWPE (B) Leaflet #2 UHMWPE + PVA; (C) Leaflet #3 UHMWPE + PVA + Collagen type I; (D) Leaflet #4 GelMA.

*Cytocompatibility of the Valcard prototype of mitral valve implant*

Following the evaluation of the leaflets, we adjusted the design parameters of the Valcard implant to account for pediatric growth, valve implant safety, and biocompatibility. These adjustments were aimed at optimizing cell viability, proliferation, and adhesion, as well as enhancing the biomaterial’s versatility for 3D design. GelMA was chosen as the biomaterial for the prototype due to its potential to enable scalability towards the development of a complete valvular model through volumetric bioprinting, followed by seeding with AoMAB cells. Cell viability was evaluated in transparent 18.4 mm-diameter Valcard implants at 14-days of culture in 12- and 24- wells (**Fig. 6**). Confocal, MesoSPIM and light sheet microscopy confirmed cell proliferation around the entire implant surface (**Movies S1-S3**).

**
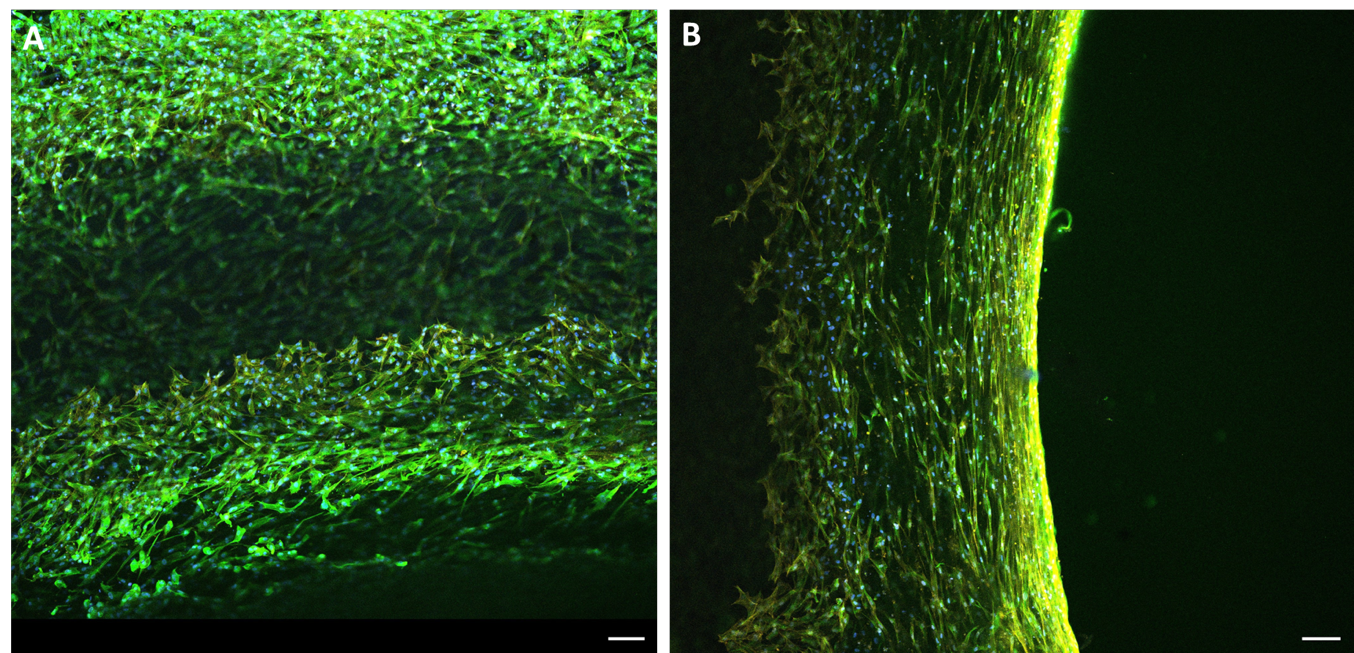
**

**Fig. 6.** Immunostaining analysis of AoMab cells on the mitral valve implant prototype Valcard. *In vitro* culture of the 3D structure implant prototype with GelMA was performed for 14 days. Green (vimentin); blue (nucleus). Scale bar: 100 µm.

**DISCUSSION**

This study evaluated a range of materials as leaflet implant candidates for infant mitral valve repair. A special consideration was thus taken for designing a structure that could synchronously grow and adapt to a growing child and demonstrate durability and strong mechanical properties [21,22]. We present here four leaflet designs for partial mitral valve repair and a mitral implant for complete valve replacement, using two different techniques. 1) The conventional freeze‒drying process consists on freezing followed by solvent sublimation of solvent to obtaining a porous structure but these have limitations for custom personalized 3D designs and mechanically resistant, such as for the full implant. Nevertheless, this method is easily manageable for the development of leaflets of different sizes for *in vitro* culture. 2) The 3D printing technique in volumetric bioprinters has the advantages of precision in size and complex and customized designs, as well as optical material transparency facilitating the evaluation of cytocompatibility by different advanced microscopy techniques [23].

A design for *in vitro* culture and growth of a leaflet or a complete valve implant would represent an advance for translational medicine in the pediatric field [24]. Current leaflet designs aim at autologous pericardial implants [18], yet the use of high-performance biomaterials such as UHMWPE [25] could be an alternative provided an appropriate coating that improve their profile [15], such as the leaflets obtained here that were characterized on the basis of their microstructural, nanostructural, physicochemical, and mechanical properties as well as cytocompatibility.

As the tested leaflet materials can be classified into two distinct categories—UHMWPE- based polymers and GelMA hydrogels—their material characteristics, the respective strengths, and even the characterization tests had distinct and variable feasibilities.

Multiple assays require GelMA to be lyophilization (used for dried bulk hydrogel) prior to experimentation (SEM, porosity), which may have affected the surface appearance of the material and pore sizing, porosity and interconnectivity. Visually, the pore size could be appears to form a non-porous structure [26], with pores potentially smaller than 10 μm, thereby limiting cell infiltration. Additionally, GelMA (leaflet #4) precluded from the characterization tests of fluid sorption, degradation, and traction due to the mechanical strength limitations [27] of the 2 mm thick construct. However, this restriction is overcome by designing greater thickness and sizes.

Leaflets coated by PVA and PVA + collagen type I improved the pore sizes thereby acquiring microstructural properties that are more culture friendly. Indeed, the coating can modify the smooth surface and nonpolar functional groups, so that UHMWPE regularly has poor adhesion and low interfacial properties [28]; PVA and collagen type I then modify the surface, as visualized by SEM, introduce polar functional chemical groups and roughness to the surface, as subsequently evidenced by FTIR and nanotopography. This explains the reduction in the contact angle of leaflets #2 and #3 and their sorption fluid capacity [28].

Both techniques of pore analysis—a traditional manual approach and a novel automated method—yield unique strengths and weaknesses. The automatic pore detection technique designed pores as nonelliptical shapes, aiming to approximate pore detection as close to the true shape as possible. While this method enabled to outline pores therefore more accurately outlined pores, the pore size (Ps) (which is typically determined through standard major and minor axis measurements) could not be calculated [29]. Nonetheless, one of the main advantages of this technique is its significantly faster analysis time. Particularly in samples with a high quantity of small pores, automatic detection is optimal because of both in terms of efficiency and ability to effectively identify small pores scattered throughout a sample. As the range of detectable pore sizes was greater with the automatic detection method, the pore area data results achieved in this manner are more accurate. This conclusion generally falls in line with the measured results, as leaflets #1 and #4—with smaller pore sizes—have areas fairly consistent across both techniques. Measurements for leaflets #2 and #3, meanwhile, are far larger in Technique 1, where a high level of small pores are likely not detected manually.

Some limitations remain, for which traditional ellipse methods are preferable. Threshold-based detection is not effective for images with uneven lighting, where shadows may be difficult to distinguish computationally from pores. Therefore, only images of surfaces with generally smooth textures are recommended for this approach based on deep learning. In an image with irregular pores and a count greater than 50, the automated technique 2 could be implemented.

The pore size of scaffold is crucial for promoting migration, cellular infiltration, vascularization, and the effective diffusion of nutrients, oxygen, and waste products [30]. The pore size of the PVA scaffolds or hydrogels has a wide range (50-650 µm) according to the results for leaflet #2, with a minimum reduction in leaflet #3 (with collagen type I) but a sufficient size for mesenchymal cells (17.9-30.4 μm) [31]. References for GelMA have an average pore size for hydrogels between 5 μm and 150 μm, according to the results obtained on leaflet #4 [32].

The percentage of degradation obtained at 14 days for UHMWPE is consistent with the resistance of this material to *in vivo* degradation. Owing to its chemical composition, the degradation rate of leaflet #2, which contains type I collagen, is greater than that of leaflet #3, which is related to the ability of collagenase to hydrolyze peptide bonds [33]. The presence of polar groups, such as hydroxyl groups (-OH) in PVA, particularly in leaflet #2 and #3, indirectly promotes the interaction with collagenase. This effect is attributed to the increased hydrophilicity of the polymer matrix, which facilitates water uptake, swelling, and potentially enhances enzymatic interactions with the material surface, even though PVA itself is not a natural substrate for collagenase.

The contact angles obtained in the UHMWPE are those expected for this material, which has more hydrophobic characteristics; however, the coating of leaflets #2 and #3 can modify this parameter and improve hydrophilicity by decreasing the contact angle, given the greater presence of polar groups in PVA [28]. This property is also evident in the fluid sorption capacity, which is much greater for the collagen-containing coating of leaflet #3, improving the interaction of the biomaterial with the aqueous medium [34].

FTIR analysis confirmed the chemical composition of each leaflet prototype, which presented typical absorption peaks according to their respective chemical compositions. The mechanical performance of the leaflet prototypes largely corresponded with findings from previous literature [35] and was critical in evaluating the potential of this study’s implant designs for an effective implementation for mitral valve repair. Macroscale mechanics of UHWMPE are complex to express generally, as a specific formulation technique plays a large role in determining these properties, as does the fact that UHMWPE is often utilized as the base of a composite with others. These results are promising and favor the use of implant coatings engineered for biocompatibility while not compromising mechanical properties in the case of PVA +collagen type I. The results for DMA are correlated with leaflets #1--#3 as thermoplastic materials.

Young's modulus is markedly different between mechanical stress tests and AFM, as these two methods operate on different scales. This is because the nanoindentation technique is not influenced by all components of the sample, allowing for the identification of the modulus of individual components [36]. However, it offers nanostructurally a key piece of data to understand the possible interaction of cells with the surface of each leaflet. Also given the hydrogel nature of the GelMA, adhesion and stiffness data differ significantly from the other prototypes [37]. The differences between leaflets #2 and #3 are striking despite the PVA coating amount of type I collagen is 100 µg/mL, which is sufficient to change the nanocharacteristics of the leaflet surface as well as shown by microstructural results.

AoMAB cells have good performance in viability test in all leaflets evaluated [7]. The fast degradation rate of leaflet #2, with a PVA coating and its hydrophilicity, could cause cells to detach from the surface and affect the cell viability count. We tend to exclude possible contamination issues as not apparent, but the different patron of adhesion between leaflets #3 and #4 may be correlate to pore size and cells in leaflet #4 (GelMA) may have migration difficulties initial post printing however the process remains feasible, as evidenced in Fig. 5. For this reason, they are preserved as concentric patrons, and the mechanical force inherent for the leaflet may affect the orientation of different molecular and cellular processes at the cellular adhesion interface at the micro- and nanoscale [38].

Finally, leaflet #3 has good microstructural, nanostructural characterisctics (pore size), as well as contact angle, fluid sorption capacity, simple mechanical strength test, nanostructural characterization, viability test, and proliferation and adhesion efficiency and other test are foreseen in the future such as permeability, thrombogenicity and in vivo testing in animal models. Similarly, mitral valve implant prototype Valcard needs specific characterization according to the ISO 5840-1:2021 standards [39], given its excellent *in vitro* performances in this study.

The durability of UHMWPE-based leaflet polymers supports their adaptability for pediatric applications, although this depends on further preclinical studies. However, based on the results presented, it can be hypothesized that these materials may offer potentially reparative performance, with growth dependent on the 3D cell-based construct. This could improve adaptability, although the disadvantage of requiring surgical reinterventions due to somatic growth would remain. In contrast, GelMA-based leaflets may offer a more regenerative approach, as their greater cellular compatibility could promote the generation of consistent valvular tissue that grows with the pediatric patient. Nevertheless, these constructs still present limitations in terms of maneuverability. Therefore, further studies are required.

**CONCLUSION**

This study engineered and characterized leaflets for in vitro culture, with potential applications in pediatric valve repair, utilizing AoMAB cells. Leaflets based on UHMWPE (Leaflet #2 and Leaflet #3) with coatings showed improvements in pore size, hydrophilicity, and cytocompatibility, while maintaining their mechanical properties and exhibiting low degradation rates. Among these, Leaflet #3 (UHMWPE coated with PVA and collagen type I) demonstrated the best performance, with *in vitro* degradation at 14 days (7.30% ± 18.71), a hydrophilic contact angle (26.13° ± 1.45), and adequate cell viability at 10 days (177.04% ± 68.92%). Leaflet #3 holds potential as a long-term and effective treatment option, inspired by the Ozaki model for valvular repair, with applications in the pediatric field, due to its anatomical adaptability through size adjustments. Nonetheless, limitations related to somatic growth remain and require further investigation.

Leaflet #4 (GelMA-based) demonstrated superior cell viability (216.77% ± 77.69%) and scalability of the design through 3D bioprinting, positioning it as a promising prototype for the future development of heart valve tissue with growth potential. Consequently, the cytocompatibility evaluation was conducted exclusively for prototype 4, as it represents the most complete implant design and fulfilled the greatest number of predefined design criteria (**Table S1**). This prototype was selected based on its use of a biocompatible material, its scalability to pediatric dimensions via bioprinting, and its enhanced capacity to support cell viability and proliferation. Additionally, this study introduced the use of AoMAB cells for TEHV applications, highlighting their proliferative potential, as demonstrated by cytocompatibility tests in 3D cultures. Nevertheless, further studies are needed in this area, including additional *in vitro* validations and thrombogenicity assessments, to advance these approaches.

**MATERIALS AND METHODS**

**Materials and methods**

*Materials:*

List of materials. **(Table S3.)**

**Prototypes and validation**

Prototype designs were developed in consideration of six main design parameters:

1. Regeneration of valve tissue: Implants were constructed from biomaterials with favorable bioactivity and chemical stability.
2. Adaptation to child growth: An initial pediatric size applicable in clinical and *in vitro* cultures was selected, with a leaflet diameter of 10 mm, for which the scalable Ozaki model was used as a reference.
3. Safety of the valve implant: Antithrombogenic biomaterials with a limited risk of immunological rejection were utilized.
4. Mechanical properties: Biomaterials with properties similar to or superior to those of native tissue in terms of traction and elastic modulus were investigated.
5. Biocompatibility: Cell viability and proliferation *in vitro* were emphasized.
6. Local implant placement: Designs allowed for implant placement by transcatheter intervention.

On the basis of these parameters, four leaflet prototypes and one mitral valve implant prototype were manufactured via conventional manufacturing and bioprinting techniques. The selection of materials and the various comparisons made during their characterization are exclusively based on the design parameters.

*Conventional manufacturing (leaflet prototype)*

A 100 µm thick UHMWPE membrane with dimensions of 1.56 mm × 1.95 mm × 1.90 mm was cut via a Trotec Laser S100 machine (GmbH, Austria). This UHMWPE membrane was tested and analyzed as Leaflet 1. Leaflet 2 was prepared from UHMWPE leaflet 1 and coated with 5% PVA. The PVA solution was prepared by slowly adding 2 g of PVA to 40 mL of distilled water and maintaining the solution at 40°C with agitation for 2 hours to ensure complete dispersion and swelling. The solution was then stirred at 90°C for 30 minutes to obtain homogeneity. The UHMWPE leaflet was immersed in the 5% PVA solution, frozen, and then freeze-dried for 24 hours at a condenser temperature of -50°C and a pressure of 1 mbar in a lyophilizer (LABCONCO, Fisher Scientific, USA). Leaflet 3 was prepared similarly but with a coating of 5% PVA mixed with 100 µg/mL collagen type I, followed by the same freezing and drying conditions as those used for leaflet 2. The samples for biological characterization were sterilized with ozone.

*3D printing*

Leaflet #4 and the mitral valve implant prototype were fabricated using a bioink composed of 7.5% w/v GelMA (methacrylated gelatin) and 1 mg/mL LAP photoinitiator, utilizing volumetric bioprinting with the Readily3D Tomolite vs. 2.0 (Lausanne, Suisse) Light doses of 180 mJ/cm² were applied for crosslinking, with exposure times calculated at 25.7 seconds. The dimensions of the bioprinted leaflets matched those of the conventionally manufactured leaflet prototype (**Fig. 1**). The bioprinted implants measured 0.5 mm × 0.1 mm × 14.3 mm × 18.4 mm and were composed of 5% GelMA photoresin. Light doses of 350 mJ/cm² and 400 mJ/cm². The implants were subsequently preserved in PBS until characterization.

**Characterization of the leaflet prototypes**

*Microstructural Study*

The pore size (Ps) of each leaflet material was measured from images obtained via a Scanning Electron Microscope FA-STE, -SU5000 – (Hitachi, High-Technologies, Tokyo, Japan) via Hitachi map 3D software. Pore size was evaluated for 1–3 images of 50 pores per implant material via ImageJ v.1.54f bundled with 64-bit Java 8. Software (Wayne, Rasband, NIH, USA) was used. The equation used to calculate Ps was as follows:

**(Eq. 1):**

$$Ps=1.5 \times2 \times\frac{\sqrt{a^{2}+b^{2}}}{2}$$

Two techniques were employed to quantify the pores for each leaflet type via ImageJ and were applied to three leaflet samples of each material. The first followed a protocol established in previous literature [29], in which 50 pores per SEM image were manually selected as elliptical approximations. The ellipse areas were determined, and the major and minor axes were measured, from which Ps was then calculated via the above formula.

The second technique sought to analyze pores by applying a threshold and particle detection algorithm in ImageJ (Wayne, Rasband, NIH, USA). Pores penetrating fully through the material were defined as regions on SEM images darker than a predefined, visually determined threshold. This threshold was kept the same for analysis of images of a given material and resulted in a binarized image of pore and nonpore regions. Images were then edited via the Analyze Particles plug-in. To encourage accurate counting, program-detected particles measuring under 100 µm were categorized as noise and therefore not included. Particles of all roundness values were considered. Details of this measurement technique are available in the supplementary materials **(Table S2).**

*In vitro biodegradation*

Enzymatic degradation was evaluated using 10 mm-diameter leaflet samples that were preweighed after being briefly wetted in PBS. Afterwards, the samples were incubated at 37°C with 0.1% collagenase type IV (>125 CDU/mg, Invitrogen) following a previously standardized concentration protocol. Degradation was assessed at five distinct time points: 1 day, 3 days, 7 days, and 14 days, with measurements performed in triplicate. Statistical analysis was conducted to evaluate the significance of the results across time points. Each time, 500 µL of 0.25 M EDTA was added to the relevant samples to stop the enzymatic reaction. Each implant was weighed on an analytical Sartorius BP211D (IG Instrument, Gesellschaft, Zürich, Switzerland), and the percentage of degradation was calculated relative to the initial sample weight.

*Contact angle*

Three samples of each leaflet were evaluated via an automated goniometer (Digidrop) and Digidrop software as well as a contact angle meter (https://gbxonline.com/) (GBX Etude des Technologies avancées) (GBX Scientific LTD, Dublin, Ireland). This test was performed at room temperature by applying four drops (4 µL) of sterilized water on the surface of each leaflet sample.

*Fluid sorption capacity*

A gravimetric-based liquid sorption assay was conducted to assess the absorbance abilities as part of the overall physicochemical characterization of the leaflets. Leaflet samples were first left for 24 hours in an oven at 60°C to promote dehydration and then weighed on an analytical balance (Sartorius BP211D; IG Instrument, Gesellschaft, Zürich, Switzerland) (calibrated to within 0.1–0.01 mg accuracy). Afterwards, 2 mL microtubes were prepared, each containing one leaflet sample and 2 mL of 0.1 м PBS (pH 7.4). The samples were stored in an incubator at 37°C, and three replicates of each material at each time point were evaluated by measuring the final weight at 20-minute intervals until 2 hours after the addition of PBS. The excess water was removed by lightly dabbing the samples on filter paper prior to the final weight measurements. The fluid sorption capacity (FSC) was calculated via the following equation (**Eq. 2.**):

FSC (%) = [(W_1_ – W_0_)/(W_0_)] × 100

*Fourier transform infrared (FTIR) spectroscopy*

Four samples of each leaflet prototype were evaluated via an FT-IR spectrometer Spectrum two, (Waltham, Massachusetts, USA). The spectra of the samples were read from 4000– 400 cm^-1^. This range was selected because it encompasses the principal functional groups of collagen (amide A, B, I, and II), UHMWPE (vinyl or acetyl groups), PVA (hydroxyl groups), and GelMA (methacrylic groups), each sample was subsequently scanned at a resolution of 4 cm^-1^/sample, with a PVA (1.0 mg) control sample used to confirm the chemical composition.

*Simple mechanical strength test*

The tensile modulus (dry) of each leaflet was obtained via a ZwickRowll universal testing machine (GmbH & Co. KG; Ulm, Germany) with 50 N capacity. The procedures followed the ISO 5840-1:2021 standards and ASTM D638 Type V [40]. A 50 N load cell calibration between 0.1–50 N was used, with a speed of 10 mm/min. Twelve dry samples per leaflet were tested at room temperature until rupture.

*Dynamic mechanical analysis (DMA)*

Leaflet samples were further analyzed using dynamic mechanical analysis (DMA) to assess material properties under dynamic conditions and identify variations in viscoelastic parameters. The prototype thicknesses were approximately 0.8 mm and 2 mm for the UHMWPE-based samples and GelMA, respectively. One at a time, the samples were loaded onto a DMA 242 E Artemis, NETZSCH compression testing. The furnace chamber temperature was set to 37°C after a heat ramp rate of 2°C/min and 20 minutes allotted for stabilization. The temperature during testing ranged from approximately 36°C to 39°C. The force track was 120% with an oscillating stress at several different frequencies—0.5 Hz, 1 Hz, 2 Hz, and 10 Hz—to determine the sample elastic storage modulus (E’), loss modulus (E’’), and damping factor (tan δ) for each material. The data were analyzed at a constant frequency of 1 Hz, and graphs were created with NETZSCH Proteus software.

*Nanostructural characterization*

Dry leaflet samples were evaluated via atomic force microscopy (AFM) Quantitative nanomechanical mapping (QNM) Bruker dimension Econ with ScanAsyst (Massachusetts, USA). Tests were conducted by tapping (contact mode) at room temperature (21ºC) a scanAsyst at resolution of 512 × 512 data points at 1 Hz via an SNL-10 tip and XE-100 model from Park System. The nanotopography, elastic modulus, adhesion, and stiffness were measured with spring constant of 0.58 nN/nm. The obtained images were analyzed via Gwyddion v2.65 software (<http://gwyddion.net/download.php)>.

*Cytocompatibility of the leaflet prototype*

AoMABs were isolated and expanded to passage 11 via a protocol described previously [8].

T25 was flask coated with 1% collagen type I (Sigma‒Aldrich). The medium used for AoMAB proliferation was prepared with 82% IMDM GlutaMAX (Gibco, 31980-030), 15% heat-inactivated FBS (A5256701), 1% penicillin/streptomycin, insulin-transferrin-selenium (100X), 1% nonessential amino acids (100X), (100 µL) 2-mercaptoethanol (100 mM), and (5 µL) human b-FGF (0.1 µg/ml). The cultures were maintained at 5000 cells per cm^2^ in a humidified incubator with 5% CO_2_ and were split every 2–3 days.

*Viability test:* AoMAB cells were seeded on 2D transparent 96-well plates (confluent after 1 day), the media were removed, and 50 µL of the mixture in each well was supplemented with Calcein-AM (Thermo Fisher, #C1430) at 1 mg/mL (5 µL), propidium iodide (Fluka, 81845) at 1 mg/mL (5 µL), Hoeschst (1X) at 0.4 µL, and DPBS (Sigma, D8537) at 1 mL. Then, the plate was incubated for 20 min at 37°C and 5% CO_2_, and the number of cells with an automated cell counter (RWD, USA) was determined. 2D AoMAB cells culture were used as a positive control.

*Cell proliferation:* Four samples of each leaflet #1--#4 prototype were sterilized with ozone SteriLux (Switzerland) for at least 15 days before the experiment. AoMAB cells (10^6^) were seeded on each leaflet (10 mm in diameter) in 1 mL of proliferation medium and were subsequently incubated (37°C, 5% CO_2_) for 2, 5 and 10 days. Each time the medium was removed from the well and washed with 1X PBS, the leaflet was incubated with 500 µL of proliferation medium and 500 µL of CyQUANT^®^ direct cell proliferation mixture for 1 h at 37°C; then, 200 µL was transferred to a 96-well plate, and the fluorescence was measured at a 508 nm peak excitation wavelength. The absorbance was obtained with a FlexStation 3 spectrophotometer (Molecular Devices; California, USA) and interpolated into a calibration curve of AoMAB cells.

*Cell adhesion:* CellTracker™ Green CMFDA C7025 (50 µg) was used to adhere adherent cells according to the manufacturer’s protocol. First, the culture media was removed, the prewarmed CellTracker working solution (10 µM) was added, and the samples were incubated for 40 minutes (37°C, 5% CO_2_). Then, the CellTracker was removed, and the proliferation medium AoMAB was added. Finally, images were obtained with an Axio Observer fluorescence microscope (ZEISS, Germany).

*Cytocompatibility of the Valcard prototype of mitral valve implant*

A 3D-printed prototype made of 5% GelMA preserved in 1X PBS was seeded with AoMAB (1x10^6^ cells/mL in 5 mL) in proliferation medium seven days after bioprinting and cultured for 14 days, after which the medium was changed every two days. Finally, the 3D cultures were fixed with 4% paraformaldehyde (PFA) for 30 minutes. Samples previously fixed were washed with PBS, and after adding 0.1% PBS-NA azide, they were preserved at 4°C for immunofluorescence.

*Immunostaining analysis:* The fixed samples were permeabilized with 0.1% Triton in 1× PBS for 30 minutes, washed with 1× PBS on a shaker 3 times for 5 minutes each, and then blocked with 1% PBS-BSA for 10 minutes at room temperature. The primary antibody mouse anti-vimentin (1:1000, ab20346, Abcam, Cambridge, MA, USA) was subsequently added to the samples, which were subsequently incubated overnight at 4°C. The secondary antibody Alexa 488-conjugated anti-mouse (1:500, A-11001, Invitrogen, Waltham, MA, USA) was subsequently added for 1 hour at 37°C. The samples were washed with 1% BSA and 0.1% BSA for 5 minutes each before being washed twice in PBS for 5 minutes. The nuclei were stained with DAPI (1:1000 Sigma‒Aldrich, 022M4004V, GmbH, München, Deutschland), and the cytoskeleton was stained with phalloidin-532. Images were acquired with a Nikon eclipse Ti2 confocal microscope (Nikon, Tokyo, Japan) with dimensions of 10×; 40×; Plan Apo objectives LWD (Nikon; Tokyo, Japan) software Nis-Elements AR 5.41.02; SPED/COLM light-sheet microscope; and MesoSPIM Olympus MVPLAPO 1 X (Olympus, Tokyo, Japan).

**Statistical analysis**

R Project statistical analysis software (R Foundation for Statistical Computing, Vienna, Austria, R version 4.0.2, 2020) and GraphPad Prism 8 were utilized (GraphPad, USA) for the analysis. The significant differences are reported at p<0.05 according to the ANOVA results (one- and two-way ANOVA).

**ACKNOWLEDGMENTS**

The principal author acknowledges support from the Federal Commission for Scholarships for Foreign Students for the Swiss Government Excellence Scholarships (ESKAS No. 2023.0458) for the academic year 2023--24, and all the authors acknowledge HEPIA for funding the materials.

The principal author acknowledges several people for their technical training and advice: from HEPIA: Dr. Professor Irena Milosevic and Mathieu Di Franco (mechanical and physicochemical component); Dr. João Marques, Dr. Schmidt Cédric (microscopy)¸; and the Tissue Engineering Laboratory team (Dr. Professor Luc Stoppini, Dr. Marc O. Heuschkel, Laetitia Nikles, Loris Gomez Baisac, Jessica Charrière and Maé Clet). From the Foundation Campus Biotech Geneva, Anthony Guillet (AFM and SEM training), Dr. Théo Ribierre (imaging), and Jeremy Laedermann (mechanical). From the Wyss Center: Dr. Laura Batti & Dr. Ivana Gantar (imaging). The DSM company provided the UHMWPE membrane. From Colombia: Alberto Benavides; Dr. Ronald Jiménez, Dra. Diana M. Millán, Dra. Adriana Flórez, María Alejandra Torres, Dra. Martha Raquel Fontanilla.

**Author contributions:** G.M.: Conceptualization, Methodology, Investigation, Visualization, Formal analysis, Data curation, Funding acquisition, Writing—original draft. R.A.: Investigation, Methodology, Writing—review & editing, funding acquisition, supervision. B.A.: Investigation and writing & editing. L.F.: Investigation. P.V.: Investigation. M.J.: Investigation; Design. S.S.: Investigation. J.M.: Writing—review & editing, funding acquisition; J.M.: Investigation. The manuscript was reviewed for all authors.

**Funding:** The principal author of this work was supported by the Federal Commission for Scholarships for Foreign Students for the Swiss Government Excellence Scholarships (ESKAS No. 2023.0458) for the academic year 2023—24.

**Competing interests:** The authors report no conflicts of interest.

**DATA AVAILABILITY**

The data that support the findings of this project are available from the corresponding author upon reasonable request.

.

SUPPLEMENTARY MATERIALS

Figures S1 to S6

Tables S1 to S3

Movies S1 to S3

REFERENCES

[1] Iddawela S, Joseph PJS, Ganeshan R, Shah HI, Olatigbe TAT, Anyu AT, Hadi K, Tarmahomed A, Harky A. Paediatric mitral valve disease - from presentation to management. Eur J Pediatr 2022;181:35–44. https://doi.org/10.1007/s00431-021-04208-7.

[2] Tuncer ON, Ertugay S, Akhundova M, Levent E, Atay Y. Long-term outcomes of mitral valve repair in children. Front Cardiovasc Med 2024;11:1454649. https://doi.org/10.3389/fcvm.2024.1454649.

[3] Van Puyvelde J, Meyns B, Rega F, Gewillig M, Eyskens B, Heying R, Cools B, Salaets T, Hellings P-W, Meuris B. Mitral valve replacement in children: balancing durability and risk with mechanical and bioprosthetic valves. Interdiscip Cardiovasc Thorac Surg 2024;38:ivae034. https://doi.org/10.1093/icvts/ivae034.

[4] Fioretta ES, Motta SE, Lintas V, Loerakker S, Parker KK, Baaijens FPT, Falk V, Hoerstrup SP, Emmert MY. Next-generation tissue-engineered heart valves with repair, remodelling and regeneration capacity. Nat Rev Cardiol 2021;18:92–116. https://doi.org/10.1038/s41569-020-0422-8.

[5] Bauser-Heaton H, Barry OM, Hofferberth SC, Tretter JT, Ma M, Goldstone A, Armstrong A, Jones TK, Yoganathan A, del Nido P. Challenges and Priorities for Children With Congenital Valvar Heart Disease: The Heart Valve Collaboratory. JACC: Advances 2024;3:101191. https://doi.org/10.1016/j.jacadv.2024.101191.

[6] Theodoris CV, Zhou P, Liu L, Zhang Y, Nishino T, Huang Y, Kostina A, Ranade SS, Gifford CA, Uspenskiy V, Malashicheva A, Ding S, Srivastava D. Network-based screen in iPSC-derived cells reveals therapeutic candidate for heart valve disease. Science 2021;371:eabd0724. https://doi.org/10.1126/science.abd0724.

[7] Ronzoni FL, Lemeille S, Kuzyakiv R, Sampaolesi M, Jaconi ME. Human fetal mesoangioblasts reveal tissue-dependent transcriptional signatures. Stem Cells Translational Medicine 2020;9:575–89. https://doi.org/10.1002/sctm.19-0209.

[8] Schwab A, Levato R, D’Este M, Piluso S, Eglin D, Malda J. Printability and Shape Fidelity of Bioinks in 3D Bioprinting. Chem Rev 2020;120:11028–55. https://doi.org/10.1021/acs.chemrev.0c00084.

[9] Filippi M, Mekkattu M, Katzschmann RK. Sustainable biofabrication: from bioprinting to AI-driven predictive methods. Trends in Biotechnology 2024;0. https://doi.org/10.1016/j.tibtech.2024.07.002.

[10] Hull SM, Lindsay CD, Brunel LG, Shiwarski DJ, Tashman JW, Roth JG, Myung D, Feinberg AW, Heilshorn SC. 3D Bioprinting using UNIversal Orthogonal Network (UNION) Bioinks. Adv Funct Mater 2021;31:2007983. https://doi.org/10.1002/adfm.202007983.

[11] Bercea M. Rheology as a Tool for Fine-Tuning the Properties of Printable Bioinspired Gels. Molecules 2023;28:2766. https://doi.org/10.3390/molecules28062766.

[12] Rodríguez-Rego JM, Mendoza-Cerezo L, Macías-García A, Marcos-Romero AC, Carrasco-Amador JP. Comparison of the potential for bioprinting of different 3D printing technologies. Int J Bioprint 2023;9:680. https://doi.org/10.18063/ijb.680.

[13] Chocholata P, Kulda V, Dvorakova J, Kolaja Dobra J, Babuska V. Biological Evaluation of Polyvinyl Alcohol Hydrogels Enriched by Hyaluronic Acid and Hydroxyapatite. Int J Mol Sci 2020;21:5719. https://doi.org/10.3390/ijms21165719.

[14] Lee JM, Suen SKQ, Ng WL, Ma WC, Yeong WY. Bioprinting of Collagen: Considerations, Potentials, and Applications. Macromolecular Bioscience 2021;21:2000280. https://doi.org/10.1002/mabi.202000280.

[15] Abdul Samad M. Recent Advances in UHMWPE/UHMWPE Nanocomposite/UHMWPE Hybrid Nanocomposite Polymer Coatings for Tribological Applications: A Comprehensive Review. Polymers 2021;13:608. https://doi.org/10.3390/polym13040608.

[16] Basir A, Grobben RB, Cramer MJ, Van Herwaarden JA, Vink A, Pasterkamp G, Kluin J, Gründeman PF. Flexible mechanoprosthesis made from woven ultra-high-molecular-weight polyethylene fibres: proof of concept in a chronic sheep model. Interactive CardioVascular and Thoracic Surgery 2017;25:942–9. https://doi.org/10.1093/icvts/ivx244.

[17] Madrid-Wolff J, Toombs J, Rizzo R, Bernal PN, Porcincula D, Walton R, Wang B, Kotz-Helmer F, Yang Y, Kaplan D, Zhang YS, Zenobi-Wong M, McLeod RR, Rapp B, Schwartz J, Shusteff M, Talyor H, Levato R, Moser C. A review of materials used in tomographic volumetric additive manufacturing. MRS Commun 2023;13:764–85. https://doi.org/10.1557/s43579-023-00447-x.

[18] Ozaki S. Ozaki Procedure: 1,100 patients with up to 12 years of follow-up. Turk Gogus Kalp Damar Cerrahisi Derg 2019;27:454. https://doi.org/10.5606/tgkdc.dergisi.2019.01904.

[19] Ozaki S, Kawase I, Yamashita H, Uchida S, Nozawa Y, Matsuyama T, Takatoh M, Hagiwara S. Aortic valve reconstruction using self-developed aortic valve plasty system in aortic valve disease. Interactive CardioVascular and Thoracic Surgery 2011;12:550–3. https://doi.org/10.1510/icvts.2010.253682.

[20] Ozaki S, Kawase I, Yamashita H, Uchida S, Nozawa Y, Takatoh M, Hagiwara S. A total of 404 cases of aortic valve reconstruction with glutaraldehyde-treated autologous pericardium. J Thorac Cardiovasc Surg 2014;147:301–6. https://doi.org/10.1016/j.jtcvs.2012.11.012.

[21] Cordoves EM, Vunjak-Novakovic G, Kalfa DM. Designing Biocompatible Tissue Engineered Heart Valves In Situ. Journal of the American College of Cardiology 2023;81:994–1003. https://doi.org/10.1016/j.jacc.2022.12.022.

[22] Ospina MY, Jiménez AN, Beltran LN, Gachancipá JF, Steffens G, González MM, González MI. Ingeniería de tejidos en población pediátrica: una esperanza para el tratamiento de enfermedades valvulares mitrales congénitas. Cirugía Cardiovascular 2023;30:141–50. https://doi.org/10.1016/j.circv.2022.10.006.

[23] Madrid-Wolff J, Toombs J, Rizzo R, Bernal PN, Porcincula D, Walton R, Wang B, Kotz-Helmer F, Yang Y, Kaplan D, Zhang YS, Zenobi-Wong M, McLeod RR, Rapp B, Schwartz J, Shusteff M, Talyor H, Levato R, Moser C. A review of materials used in tomographic volumetric additive manufacturing. MRS Commun 2023;13:764–85. https://doi.org/10.1557/s43579-023-00447-x.

[24] Li RL, Sun M, Russ JB, Pousse P-L, Kossar AP, Gibson I, Paschalides C, Herschman AR, Abyaneh MH, Ferrari G, Bacha E, Waisman H, Vedula V, Kysar JW, Kalfa D. In Vitro Proof of Concept of a First-Generation Growth-Accommodating Heart Valved Conduit for Pediatric Use. Macromol Biosci 2023;23:e2300011. https://doi.org/10.1002/mabi.202300011.

[25] Zhou H, Wu Q, Wu L, Zhao Y. Mechanical behaviors of high-strength fabric composite membrane designed for cardiac valve prosthesis replacement. Journal of the Mechanical Behavior of Biomedical Materials 2023;142:105863. https://doi.org/10.1016/j.jmbbm.2023.105863.

[26] Li J, Mooney DJ. Designing hydrogels for controlled drug delivery. Nat Rev Mater 2016;1:1–17. https://doi.org/10.1038/natrevmats.2016.71.

[27] Guo A, Zhang S, Yang R, Sui C. Enhancing the mechanical strength of 3D printed GelMA for soft tissue engineering applications. Mater Today Bio 2023;24:100939. https://doi.org/10.1016/j.mtbio.2023.100939.

[28] Chhetri S, Sarwar A, Steer J, Dhib R, Bougherara H. Design of a bi-layer coating configuration on ultra-high molecular weight polyethylene (UHMWPE) fibre surface to derive synergistic response on interfacial bond strength. Composites Part A: Applied Science and Manufacturing 2022;152:106678. https://doi.org/10.1016/j.compositesa.2021.106678.

[29] Suesca E, Dias AMA, Braga MEM, de Sousa HC, Fontanilla MR. Multifactor analysis on the effect of collagen concentration, cross-linking and fiber/pore orientation on chemical, microstructural, mechanical and biological properties of collagen type I scaffolds. Mater Sci Eng C Mater Biol Appl 2017;77:333–41. https://doi.org/10.1016/j.msec.2017.03.243.

[30] Mukasheva F, Adilova L, Dyussenbinov A, Yernaimanova B, Abilev M, Akilbekova D. Optimizing scaffold pore size for tissue engineering: insights across various tissue types. Front Bioeng Biotechnol 2024;12. https://doi.org/10.3389/fbioe.2024.1444986.

[31] Ge J, Guo L, Wang S, Zhang Y, Cai T, Zhao RCH, Wu Y. The size of mesenchymal stem cells is a significant cause of vascular obstructions and stroke. Stem Cell Rev Rep 2014;10:295–303. https://doi.org/10.1007/s12015-013-9492-x.

[32] Miri AK, Hosseinabadi HG, Cecen B, Hassan S, Zhang YS. Permeability mapping of gelatin methacryloyl hydrogels. Acta Biomaterialia 2018;77:38–47. https://doi.org/10.1016/j.actbio.2018.07.006.

[33] Tryggvason K, Huhtala P, Höyhtya M, Hujanen E, Hurskainen T. 70 K type IV collagenase (gelatinase). Matrix Suppl 1992;1:45–50.

[34] He J, Yu H, Wang L, Yang J, Zhang Y, Huang W, Ouyang C. Hygroscopic photothermal sorbents for atmospheric water harvesting: From preparation to applications. European Polymer Journal 2024;202:112582. https://doi.org/10.1016/j.eurpolymj.2023.112582.

[35] Hussain M, Naqvi RA, Abbas N, Khan SM, Nawaz S, Hussain A, Zahra N, Khalid MW. Ultra-High-Molecular-Weight-Polyethylene (UHMWPE) as a Promising Polymer Material for Biomedical Applications: A Concise Review. Polymers (Basel) 2020;12:323. https://doi.org/10.3390/polym12020323.

[36] Sadeghpour F, Darkhal A, Gao Y, Motra HB, Aghli G, Ostadhassan M. Comparison of geomechanical upscaling methods for prediction of elastic modulus of heterogeneous media. Geoenergy Science and Engineering 2024;239:212915. https://doi.org/10.1016/j.geoen.2024.212915.

[37] Bova L, Maggiotto F, Micheli S, Giomo M, Sgarbossa P, Gagliano O, Falcone D, Cimetta E. A Porous Gelatin Methacrylate-Based Material for 3D Cell-Laden Constructs. Macromolecular Bioscience 2023;23:2200357. https://doi.org/10.1002/mabi.202200357.

[38] Tang VW. Cell–cell adhesion interface: orthogonal and parallel forces from contraction, protrusion, and retraction. F1000Res 2018;7:F1000 Faculty Rev-1544. https://doi.org/10.12688/f1000research.15860.1.

[39] ISO 5840-1:2021. Implants cardiovasculaires — Prothèses valvulaires — Partie 1: Exigences générales 2021.

[40] ASTM International D638-22. ASTM D638-22. Standard Test Method for Tensile Properties of Plastics 2022. https://www.astm.org/d0638-22.html (accessed August 20, 2024).

**Supplementary materials**

Tissue Engineering *in vitro* Leaflet and 3D Printing-Based Implant Prototypes for Infant Mitral Valve.

Martha I. González-Duque^1,2,3^, Arielle Breuninger^1^, Frédéric Leis^4^, Julio B. Michaud^1^, Shaginth Sivakumar^1^, Vincent Pautu^5^, Marisa E. Jaconi^4^, Marc Jobin^5^ and Adrien Roux^1^*

^1.^ Tissue Engineering Laboratory, Bioengineering Group, HEPIA HES-SO University of Applied Sciences and Arts Western Switzerland, Geneva, Switzerland.

^2.^ Tissue Engineering Group, Departamento de Farmacia, Facultad de Ciencias, Universidad Nacional de Colombia, Av. Carrera 30 # 45-10, Bogotá 111321, D.C., Colombia.

^3.^ Biomedical Sciences Group, Department of Medicine, Universidad Antonio Nariño. Carrera 1 #47a – 15, Bogotá D.C., Colombia.

^4.^ Department of Basic Neurosciences, Faculty of Medicine, University of Geneva, Switzerland.

^5.^ Materials, Optics and Nanotechnology Group, HEPIA HES-SO University of Applied Sciences and Arts Western Switzerland, Geneva, Switzerland.

*Address correspondence to: Adrien Roux; [adrien.roux@hesge.ch](mailto:adrien.roux@hesge.ch)


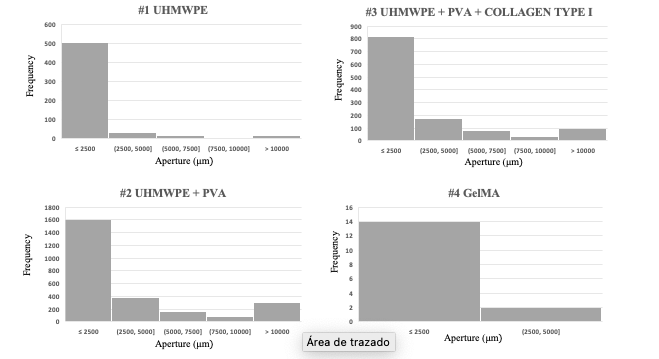


**Fig. S1.** Histogram showing the distribution of aperture sizes for Leaflet #1 UHMWPE; Leaflet #2 UHMWPE+PVA 5%; Leaflet #3 UHMWPE+PVA 5%+100 µg/mL collagen type I; Leaflet #4 GelMA 7.5% w/v.


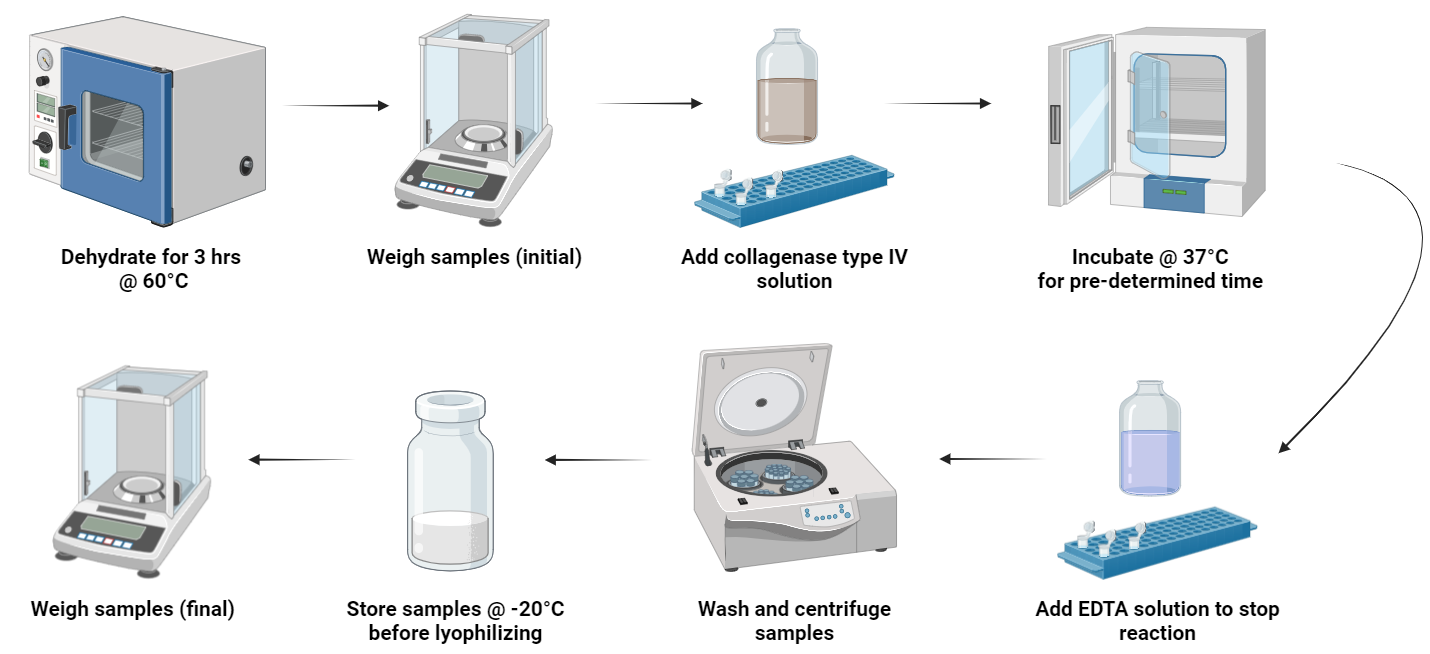


**Fig. S2.** Biodegradation assay protocol outline.


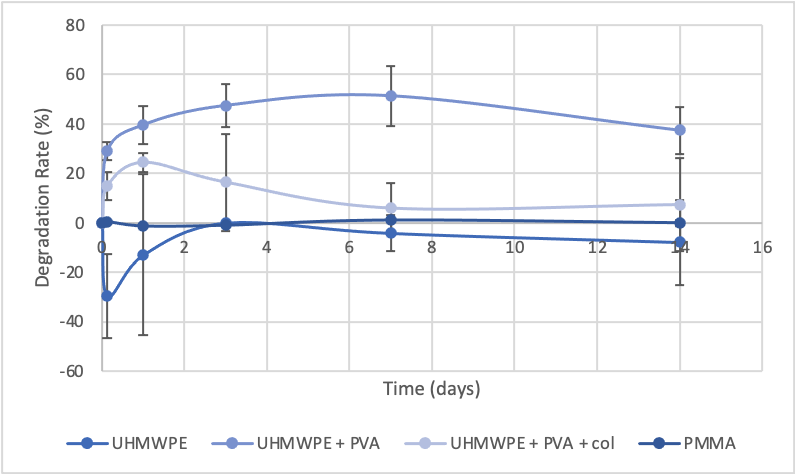


**Fig. S3.** Biodegradation assay results.


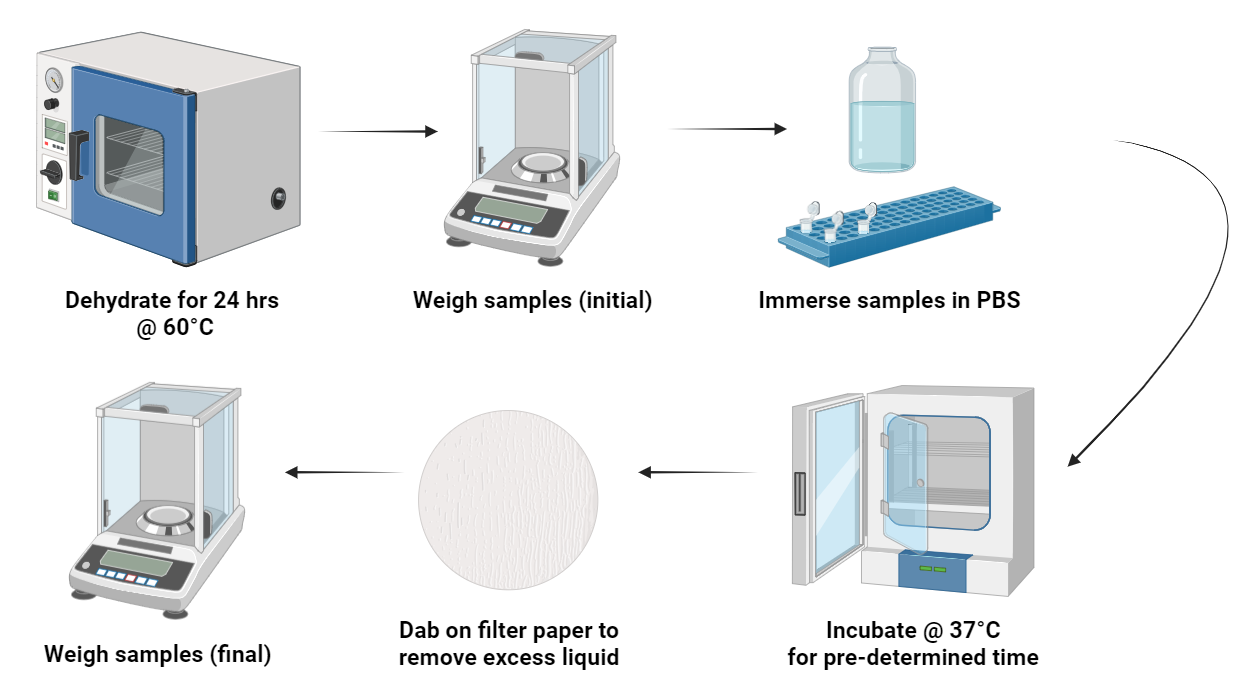


**Fig. S4**. Fluid sorption capacity assay protocol outline.

**
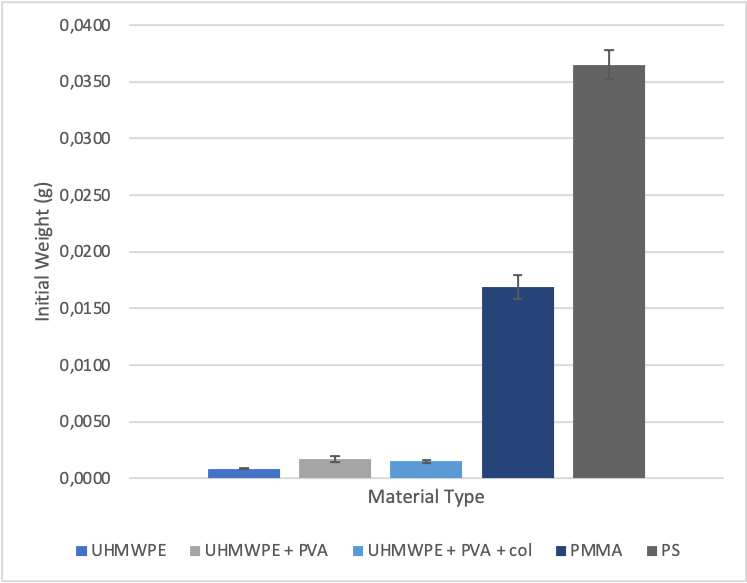
**

**N = 18**

**Fig. S5.** Fluid sorption capacity assay results of initial weight

**
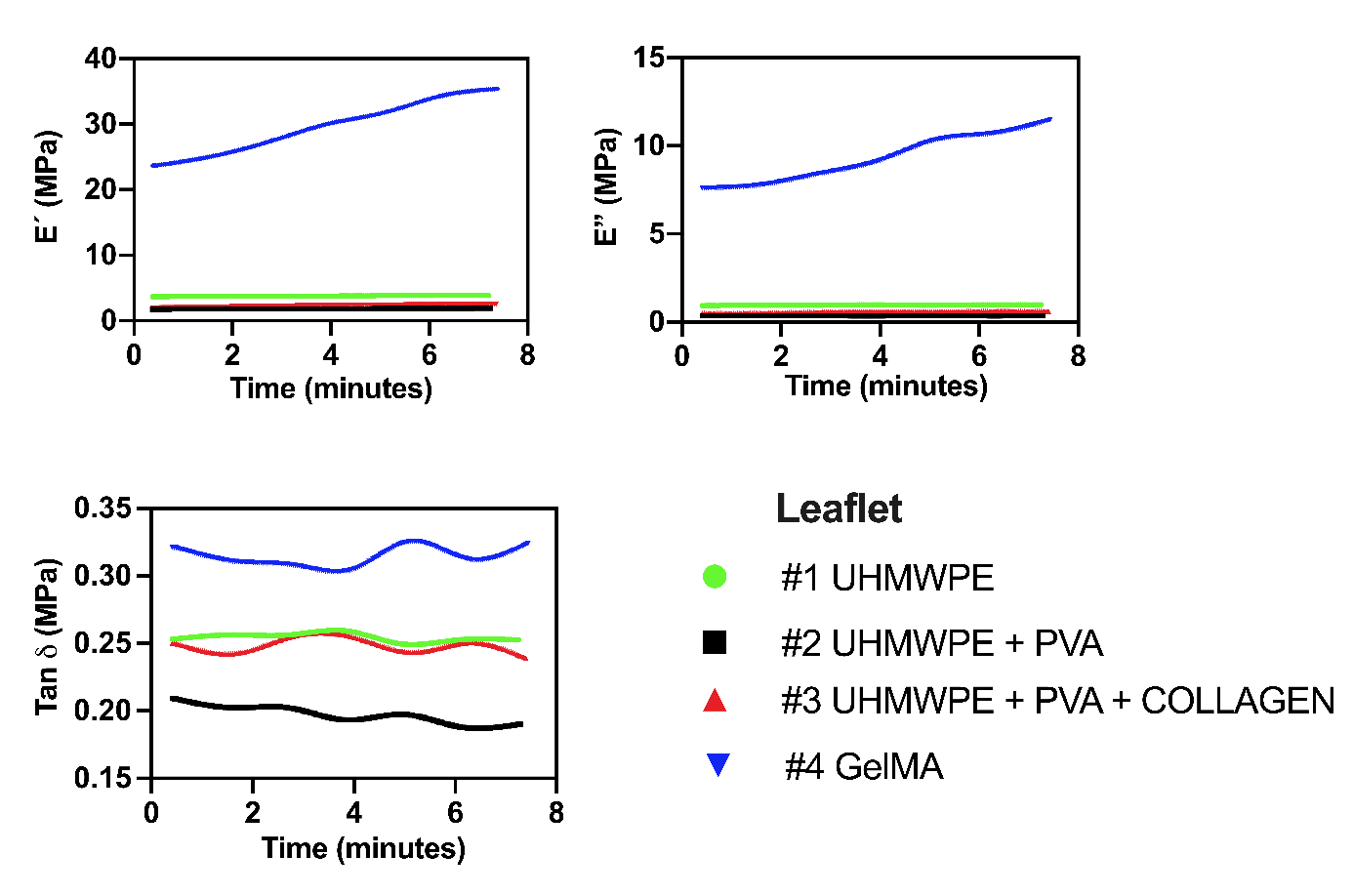
 Fig. S6.** Dynamic mechanical analysis (DMA). Images of leaflets #1--#4. Leaflet #1 (UHMWPE); leaflet #2 (UHMWPE + PVA); leaflet #3 (UHMWPE + PVA + collagen type I); leaflet #4 (GelMA). storage (E’) and loss (E”) moduli and the loss factor (tan δ) at 1 Hz.


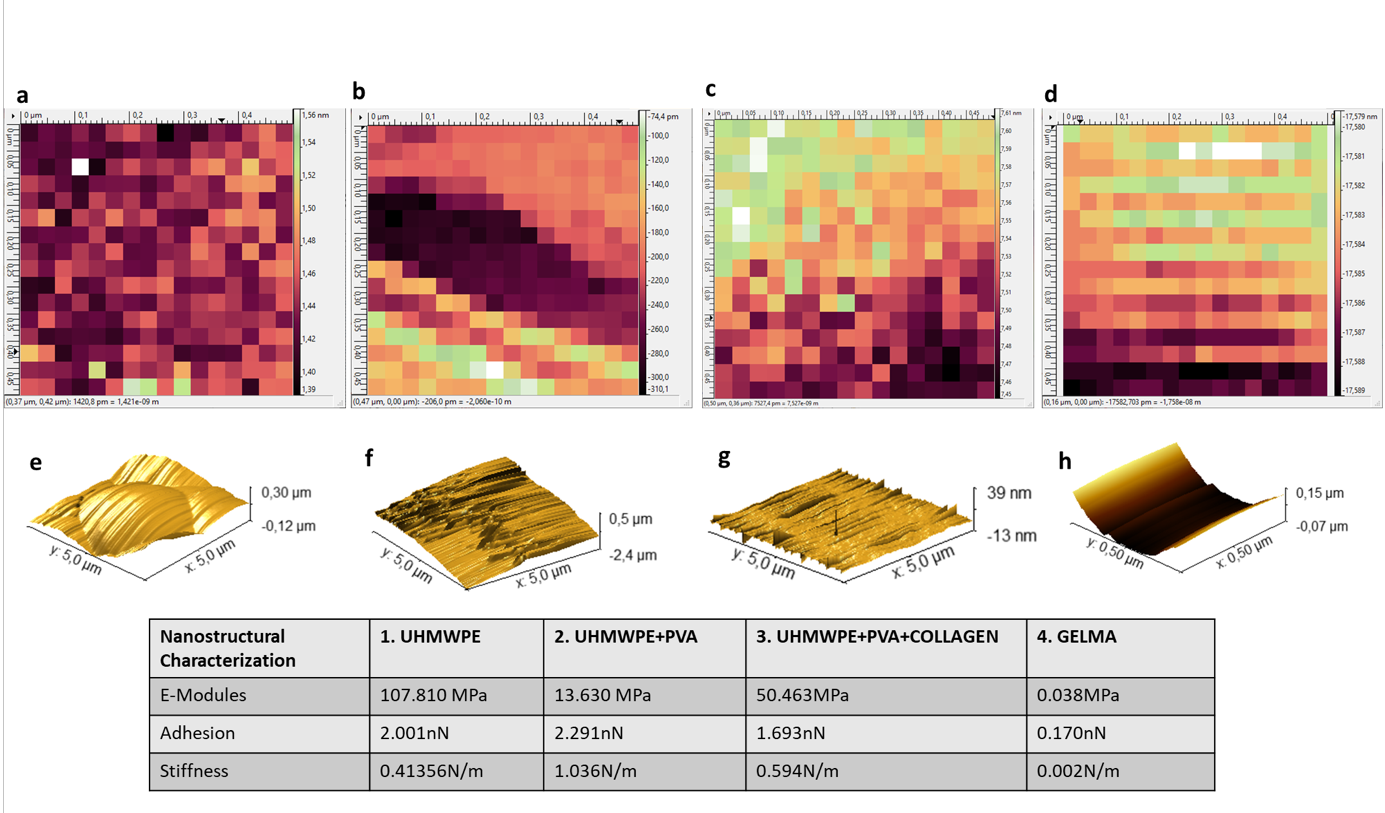


**Fig. S7.** Nanostructural characterization of the leaflet prototypes. (a-d) Elastic modulus force maps (16 × 16 pixels) from each leaflet. (a) Leaflet #1 UHMWPE (b) Leaflet #2 UHMWPE + PVA (c) Leaflet #3 UHMWPE + PVA + Collagen type I (d) Leaflet #4 GelMA. (e-h) AFM topography images of a 5 × 5 µm area; (e) Leaflet #1 UHMWPE; (f) Leaflet #2 UHMWPE + PVA; (g) Leaflet #3 UHMWPE + PVA + Collagen type I; (h) Leaflet #4 GelMA. Values for nanostructural characterization: E-modules: megapascal (MPa); Adhesion: nano-Newtons (nN); Stiffness: Newtons per meter (N/m). Leaflet # 1 UHMWPE, Leaflet #2UHMWPE + PVA, Leaflet #3 UHMWPE + PVA + Collagen type I, Leaflet #4 GelMA.

**Fig. S8.** Quantification of the fluorescence intensity from each leaflet. (1) Leaflet #1 UHMWPE, (2) Leaflet #2 UHMWPE + PVA (3) Leaflet #3 UHMWPE + PVA + Collagen type I (4) Leaflet #4 GelMA. Control: unseeded leaflet. n = 3; mean= SD; *p ≤ 0.05.


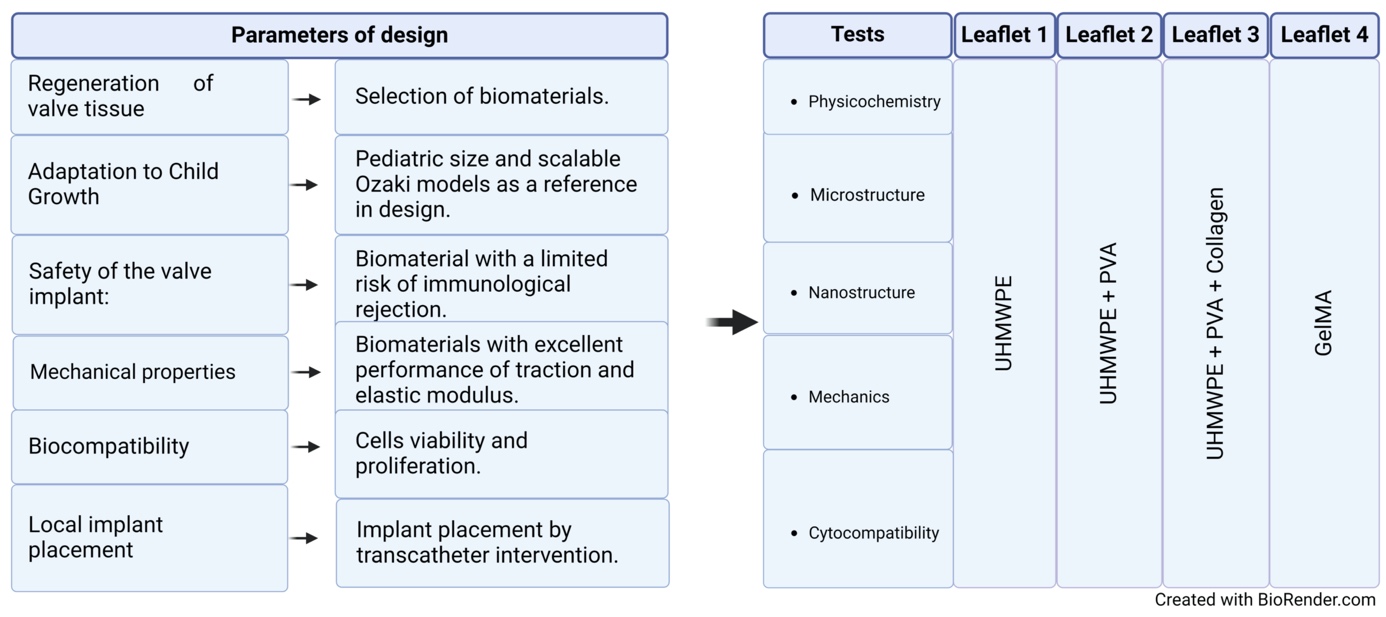


**Table S1.** Parameters of design for experimental setup validation

| **Leaflet 1 SEM images** | **Leaflet #2 and Leaflet #3 SEM images** | **Leaflet #4 SEM images** |
| --- | --- | --- |
| 1.The scale of the image is set to convert from pixels to µm. | 1.The scale of the image is set to convert from pixels to µm. | 1.The scale of the image is set to convert from pixels to µm. |
| 2.Crop to remove the scale bar from the analyzed image. | 2.Crop to remove the scale bar from the analyzed image. | 2.Crop to remove the scale bar from the analyzed image. |
| 3.The image contrast is adjusted such that the minimum and maximum values are set to 78 and 235, respectively. | 3.A threshold is applied to the image. Set settings to Default, Over/Under, and apply Dark Background. The threshold bars are adjusted to 36 and 255. | 3.A threshold is applied to the image. Set settings to Default, Over/Under, and apply Dark Background. The threshold bars are adjusted to 78 and 255. |
| 4.A threshold is applied to the image. Set settings to Default, Over/Under, and apply Dark Background. The threshold bars are adjusted to 36 and 255. | 4.Despeckle the binarized image produced above. | 4.Despeckle the binarized image produced above. |
| 5.“Despeckle” the binarized image produced above. | 5.Invert the image via look-up tables. | 5.Invert the image using Look-up Tables. |
| 6.Invert the image via look-up tables. | 6.A watershed was applied to the image. | 6.Apply Watershed to the image. |
| 7.A watershed was applied to the image. | 7.Run the Analyze Particles program. The size range is set to 1–2000 µm, the circularity range is 0.00–1.00, and overlay masks are shown. | 7.Run the Analyze Particles program. Set size range to 12-1000 µm, circularity range to 0.77-1.00, and show overlay masks. |
| 8.Run the Analyze Particles program. Run the Analyze Particles program. The size range is set to 1.00–2000 µm, the circularity range is 0.00–1.00, and overlay masks are shown. |  |  |

**Table S2.** Details of Technique 2 ImageJ protocol for pore size analysis.

| **Product** | **Reference** | **Brand** |
| --- | --- | --- |
| FBS heat‐inactivated | 10270 | Gibco, Thermo Fisher Scientific (Waltham, Massachusetts, USA) |
| Penicillin/streptomycin | 15070‐063 | Gibco, Thermo Fisher Scientific (Waltham, Massachusetts, USA) |
| Non‐essential amino acids (100X) | 11140035 | Gibco, Thermo Fisher Scientific (Waltham, Massachusetts, USA) |
| 2‐mercaptoethanol (100 mM) | M6250 | Sigma‒Aldrich, Merck, GmbH, München, Deutschland) |
| Human b‐FGF (0.1 mg/ml)^1^ | PHG0369 | Gibco, Thermo Fisher Scientific (Waltham, Massachusetts, USA) |
| Phosphate Buffered Saline (PBS) | A0965 | Applichem GmbH, Darmstadt, Deutschland |
| Methacrylated gelatin (PhotoGel® 95% DS) | 5208 | Cellink,Göteborg, Suède. |
| Photoinitiator LAP (Lithium-Phenyl-2,4,6-trimethylbenzoylphosphinat ≥95%) | 900889-1G | Sigma‒Aldrich, Merck, GmbH, München, Deutschland |
| Collagenase type IV | 17104019 | Gibco, Thermo Fisher Scientific (Waltham, Massachusetts, USA) |
| Primary human fetal mesoangioblasts (AoMABs) |  |  |
| Triton X100 | 93418 | Fluka, Thermo Fisher Scientific (Waltham, Massachusetts, USA) |
| BSA | 9048-46-8 | Sigma‒Aldrich, Merck, GmbH, München, Deutschland) |
| mouse anti-vimentin (1:1000) | ab20346 | Abcam, Cambridge, Massachusetts, USA |
| Alexa fluor 488 anti-mouse (1:500) | A-11001 | Invitrogen, Waltham, Massachusetts, USA |
| Collagen I rat tail 3 mg/Ml | A10483-01 | Gibco, Thermo Fisher Scientific (Waltham, Massachusetts, USA) |
| Poly(vinylalkohol) Mw 89,000-98,000, 99+% hydrolyzed | 341584 | Sigma‒Aldrich, Merck, GmbH, München, Deutschland |
| DAPI (1:1000) | 022M4004V | Sigma‒Aldrich, Merck, GmbH, München, Deutschland |
| Phalloidin-532 . | A22282 | Molecular Probes, Thermo Fisher Scientific, Waltham, Massachusetts, USA) |
| EDTA | A2937.0500 | Applichem GmbH, Darmstadt, Deutschland |
| Human b‐FGF (0.1 mg/ml)^1^ | 78003.1 | Stemcell technologies, Vancouverm Canada |
| Rapiclear 1.47 | #RC147001 | SUNJin Lab, Taiwan (165 Unit/10 mL) |
| Ulteeva PurityTM membrane (nominal thickness 100 µm) | A4 | dsm-firmeninch (Born, NLD) |
| IMDM Glutamax | 31980-030 | Gibco, Thermo Fisher Scientific (Waltham, Massachusetts, USA) |
| Insulin-Transferrin-Selenium (100X) | 41400045 | Gibco, Thermo Fisher Scientific (Waltham, Massachusetts, USA) |
| Nunc™ EasYFlask™ Cell Culture Flasks T25 | 156340 | Thermo Fisher Scientific (Waltham, Massachusetts, USA) |
| 96 Well Black Bottom Plate | 655090 | Greiner Bio-One International GmbH (Frickenhausen, Deutschland) |
| Calcein-AM | 17783 | Thermo Fisher Scientific (Waltham, Massachusetts, USA) |
| Propidium iodide | 81845 | Fluka, Thermo Fisher Scientific (Waltham, Massachusetts, USA) |
| Hoeschst 1X | 33342 | Invitrogen, Waltham, Massachusetts, USA |
| DPBS | D8537 | Sigma‒Aldrich, GmbH, München, Deutschland (57.10 Unit) |
| CyQUANT® direct cell proliferation assay | C35011 | Molecular Probes, Thermo Fisher Scientific, Waltham, Massachusetts, USA) |
| CellTracker™ Green CMFDA | C2925 | Invitrogen, Waltham, Massachusetts, USA |
| Paraformaldehyde PFA 4% | 16005H | Fluka, Thermo Fisher Scientific (Waltham, Massachusetts, USA) |
| Sodium azide | 13412 | Riedel-de Haen, GmbH, Seelze, Germany |

**Table S3.** List of materials. Reagents and cell sources.

**Movies.** Cytocompatibility of the mitral valve implant prototype Valcard

**Movie S1.** Confocal microscopy (Supplementary material Video 1)

<https://drive.switch.ch/index.php/s/FsCoMJRV6hwRPMM>

Immunostaining analysis of AoMab cells on the mitral valve implant prototype Valcard. *In vitro* culture of the 3D structure implant prototype with GelMA was performed for 14 days. Green (Vimentin); blue (nucleus). Magnification 40×. Scale bar: 100 µm.

**Movie S2.** Light sheet fluorescence microscopy (Supplementary material Video 2)

<https://drive.switch.ch/index.php/s/FsCoMJRV6hwRPMM>

Immunostaining analysis of AoMab cells on the mitral valve implant prototype Valcard. *In vitro* culture of the 3D structure implant prototype with GelMA was performed for 14 days. Green (Vimentin); blue (nucleus). Scale bar: 100 µm.

**Movie S3.** MesoSPIM (mesoscale selective plane illumination microscopy) (Supplementary material Video 3).

<https://drive.switch.ch/index.php/s/FsCoMJRV6hwRPMM>

Immunostaining analysis of AoMab cells on the mitral valve implant prototype Valcard. *In vitro* culture of the 3D structure implant prototype with GelMA was performed for 14 days. Green (Vimentin); blue (nucleus). Scale bar: 100 µm.
